# The Transcriptional Regulator TfmR Directly Regulates Two Pathogenic Pathways in *Xanthomonas oryzae* pv. *oryzicola*

**DOI:** 10.3390/ijms25115887

**Published:** 2024-05-28

**Authors:** Zheng Chang, Zengfeng Ma, Qian Su, Xinqi Xia, Wenxin Ye, Ruifang Li, Guangtao Lu

**Affiliations:** 1 State Key Laboratory for Conservation and Utilization of Subtropical Agro-Bioresources, College of Life Science and Technology, Guangxi University, Nanning 530004, China; 2008401017@st.gxu.edu.cn (Z.C.); 2208401009@st.gxu.edu.cn (Q.S.); 2208391038@st.gxu.edu.cn (X.X.); 1808301061@st.gxu.edu.cn (W.Y.); 2 Plant Protection Research Institute, Guangxi Academy of Agricultural Science/Key Laboratory of Green Prevention and Control on Fruits and Vegetables in South China Ministry of Agriculture and Rural Affairs/Guangxi Key Laboratory of Biology for Crop Diseases and Insect Pests, Nanning 530007, China; 3 Rice Research Institute, Guangxi Academy of Agricultural Sciences, Nanning 530007, China; mazengfeng@gxaas.net

**Keywords:** *Xanthomonas*, transcriptional regulator, virulence, RpfG, HrpX

## Abstract

*Xanthomonas oryzae* pv. *oryzicola* (*Xoc*) is a notorious plant pathogen. Like most bacterial pathogens, *Xoc* has evolved a complex regulatory network to modulate the expression of various genes related to pathogenicity. Here, we have identified TfmR, a transcriptional regulator belonging to the TetR family, as a key player in the virulence mechanisms of this phytopathogenic bacterium. We have demonstrated genetically that *tfmR* is involved in the hypersensitive response (HR), pathogenicity, motility and extracellular polysaccharide production of this phytopathogenic bacterium. Our investigations extended to exploring TfmR’s interaction with RpfG and HrpX, two prominent virulence regulators in *Xanthomonas* species. We found that TfmR directly binds to the promoter region of RpfG, thereby positively regulating its expression. Notably, constitutive expression of RpfG partly reinstates the pathogenicity compromised by TfmR-deletion mutants. Furthermore, our studies revealed that TfmR also exerts direct positive regulation on the expression of the T3SS regulator HrpX. Similar to RpfG, sustained expression of HrpX partially restores the pathogenicity of TfmR-deletion mutants. These findings underscore TfmR’s multifaceted role as a central regulator governing key virulence pathways in *Xoc*. Importantly, our research sheds light on the intricate molecular mechanisms underlying the regulation of pathogenicity in this plant pathogen.

## 1. Introduction

The genus *Xanthomonas* comprises an important group of Gram-negative phytopathogenic bacteria that cause severe diseases in many important crops [1,2]. Among the most prominent of these pathogens is the species *Xanthomonas oryzae* pv. *oryzicola* (*Xoc*), the causal agent of bacterial leaf streak in rice (BLS), a bacterial disease that is a serious threat to rice productivity [3].

In recent years a large number of *Xoc* virulence factors have been identified and characterized. *Xoc* has been shown to produce and secrete a number of factors to promote its pathogenicity, including the extracellular polysaccharide (EPS), adhesion, diffusible signal factor (DSF) cell–cell signals, extracellular enzymes, and type III effectors (T3SEs) secreted through the type III secretion system (T3SS) et al. [3,4,5,6,7,8,9,10,11,12]. However, the molecular mechanisms that underpin the regulation of these virulence factors in *Xoc* are less explored and understood. Currently, the main pathogenic pathogenesis-related genes that have been identified in *Xoc* include the *rpf* gene and the T3SS gene, among others [5,6].

The *rpf* gene cluster in *Xoc* mainly encodes the proteins RpfF, RpfC and RpfG. In the genus *Xanthomonas* they are associated with a regulatory system involving the diffusible signaling factor (DSF) [7]. RpfF is involved in the synthesis of DSF, which is important for intercellular signaling [7,8,9]. RpfC and RpfG constitute a two-component regulatory system, and they are responsible for the perception of DSF signaling molecules and for signaling [7]. When RpfC senses DSF signaling, it undergoes self-phosphorylation and passes the phosphate group to the RpfG [7]. RpfG has a two-component system of a receptive domain and an HD-GYP structural domain [7,10]. Phosphorylation of RpfG activates its HD-GYP structural domain, which possesses the phosphodiesterase activity that hydrolyzes cyclic di-GMP, leading to changes in the intracellular level of cyclic di-GMP [7,11]. In *Xoc*, RpfG positively regulates pathogenicity and EPS production, and negatively regulates T3SS gene expression [6]. Despite the above studies on the function of RpfG, few transcription factors that directly regulate RpfG have been studied in *Xoc*.

T3SS plays a crucial role in both plant and animal pathogens successfully infected into the host [12,13]. As with many other bacterial pathogens, T3SS, which is encoded by a cluster of over 20 hypersensitive response and pathogenicity (*hrp*) genes, is an essential virulence determinant of *X*. *oryzae* [14,15]. Mutations in the T3SS gene in *X*. *oryzae* result in loss of the ability to elicit its hypersensitive response (HR) in the nonhost plant *Nicotiana benthamiana*. And the T3SS gene in *X*. *oryzae* is essential for the pathogenicity of the host rice plant [14]. In basic culture (XOM2), the T3SS gene is activated [16,17]. Notably the T3SS gene is directly activated by the AraC family transcriptional regulator HrpX in *X*. *oryzae* [18]. And the OmpR family regulator HrpG positively regulates HrpX transcription directly[17,19]. Although there are regulatory proteins that directly positively regulate HrpG/HrpX in *Xanthomonas* spp., there are also other transcription factors that have not yet been identified [20,21].

Several other regulatory proteins have been shown to control the expression of virulence factor genes in other *Xanthomonas* species. TfmR is a transcriptional regulator belonging to the TetR family of transcriptional regulators (TFRs). In *Xanthomonas citri* subsp. *citri* (*Xcci*) strain 306, TfmR indirectly regulates the expression of T3SS genes through the direct regulation of fatty acid metabolism genes [22]. TfmR plays a positive role in the pathogenicity, HR, motility, and production of EPS in the *Xcci* strain 306 [22]. TFRs are usually used as transcriptional repressors to regulate the expression of target gene expression [23]. Their regulatory functions vary widely and are now found to be related to bacterial drug resistance, community sensing, catabolism, antibiotic production, and host response [24,25].

Despite the above advances in TfmR in the *Xcci 306* strain, little is known about the function of TfmR in *Xoc*. Too investigate its role in *Xoc*, we knocked out TfmR in the *Xoc* GX01 strain. We found that the pathogenicity of the EPS production, motility, HR, and proliferation in the host plant of the deletion mutant Δ*tfmR* were significantly attenuated. 

Interestingly, the expression of *rpfG* in the Δ*tfmR* mutant restores motility and EPS yield, and partially restores the pathogenicity and proliferative capacity of the mutant in host plants. In addition, through a series of experiments we showed that TfmR can directly bind to the promoter region of *rpfG* and positively regulate the expression of the *rpfG* gene. Our experimental evidence indicates that TfmR can directly bind to the *hrpX* promoter region and activate its gene expression, and that HrpX restores the mutant’s HR and partially restores pathogenicity and proliferative capacity in the host plant. Based on our evidence, we can show that in *Xoc*, TfmR regulates a series of cellular processes through the regulation of RpfG and HrpX, respectively, which in turn regulates pathogenicity in the host plant.

## 2. Results

### 2.1. TfmR Plays a Role in Xoc Full Virulence

To explore the role of TetR family transcriptional regulators in *Xoc*, we conducted sequence comparison and protein sequence-identity analysis of all nine TetR family transcriptional regulators in *Xoc*. The analysis showed low sequence identity of these proteins. (Figure S1A,B), suggesting potential functional diversity in *Xoc*.

Subsequently, we generated deletion mutants for each of the nine TetR family transcriptional regulators using the homology double-swap method. These mutants were then inoculated into rice (*Oryza sativa* L. ssp. *Japonica* cultivar *Nipponbare*) using the leaf-infiltration method (Figure S1C). Our results indicated a significant reduction in pathogenicity, specifically in the mutant strain ΔXOCgx_1556. Remarkably, the amino acid sequence encoded by *XOC*gx_1556 (accession number QEO96549.1) shared a high similarity (98.5%) with the *Xcci* TfmR (strain 306) [22]. Consequently, we designated the protein encoded by XOCgx_1556 as TfmR.

**Figure 1:**
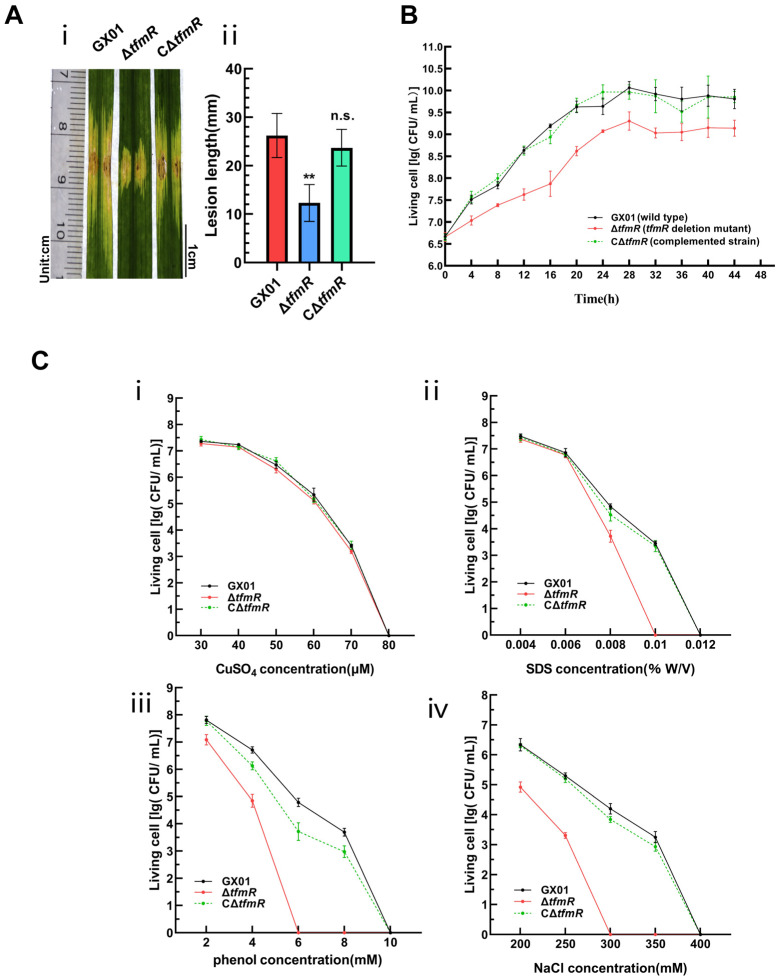
TfmR is required for full virulence of *Xoc*. (**A**) *Xoc* strain pathogenicity assay. (**i**) Phenotypic chart of lesion length. (**ii**) Statistical chart of lesion length, values are means ± SD (*n* = 30). Significance was determined by ANOVA and Dunnett’s post hoc test to compare with the wild type. ** *p* < 0.01; n.s., not significant. The experiment was repeated three times with similar results. (**B**) Growth of *Xoc* strains in complex medium (NB). The strains were inoculated into NB medium at a final density of 0.01 (OD_600_). The growth of the strains was recorded every 4 h. (**C**) Stress tolerance test of *Xoc* strains. Survival experiments performed by subculturing strains overnight on fresh NA plates supplemented with different concentrations of CuSO_4_ (**i**), SDS (**ii**), phenol (**iii**), and NaCl (**iv**). Values given are the means ± SD (*n* = 3 biological repeats).

To further elucidate the function of TfmR in *Xoc*, we utilized the pXUK [26] plasmid to construct the complementary strain CΔ*tfmR*. Pathogenicity assays were performed on wild-type strain GX01, deletion mutant Δ*tfmR*, and complementation strain CΔ*tfmR*. Lesions induced by Δ*tfmR* were significantly shorter compared to the wild type (Figure 1A). Notably, the complemented strain CΔ*tfmR* exhibited virulence symptoms (lesion length) similar to those of the wild type (Figure 1A and Figure S3A).

To investigate the specific functions of TfmR in manipulating *Xoc* pathogenesis, we conducted a series of phenotypic tests. Compared to the wild type, the growth rate of the deletion mutant Δ*tfmR* in nutrient-rich liquid medium (NB) significantly lagged behind during the early exponential phase (Figure 1B). Notably, the growth status of the complementation strain CΔ*tfmR* in NB medium remained consistent with that of the wild type (Figure 1B), suggesting an indispensable role of TfmR in *Xoc* growth. Furthermore, our results demonstrated that the deletion mutant Δ*tfmR* exhibited a lower survival rate under stress conditions induced by sodium dodecyl sulfate (SDS), phenol, and NaCl, while the complemented strain responded similarly to the wild type (Figure 1C). Additionally, the secretion of protease and amylase was higher in the deletion mutant Δ*tfmR* compared to the wild type, and in the complemented strain, the enhanced enzyme activities were restored to wild-type levels (Figure S4).

These findings suggest that in *Xoc*, TfmR has a key role in bacterial growth and confers resistance to specific stressors, but appears to negatively regulate extracellular enzyme production. However, despite these findings, the regulatory mechanisms of TfmR in *Xoc* pathogenicity remain unclear.

### 2.2. TfmR Is a Global Regulator That Regulates the Expression of a Large Number of Genes Involved in Various Cellular Processes

To further elucidate the regulatory role of TfmR in *Xoc*, we conducted RNA sequencing (RNA-Seq) to determine the transcriptome of the TfmR-deletion mutant strain Δ*tfmR*. Both the mutant strain and the wild-type strain GX01 were cultured in NB medium until reaching the mid-exponential phase (OD_600_ = 0.8). Subsequently, bacterial RNA was extracted, library preparation was performed, and the generated data were analyzed for differential gene expression.

**Figure 2:**
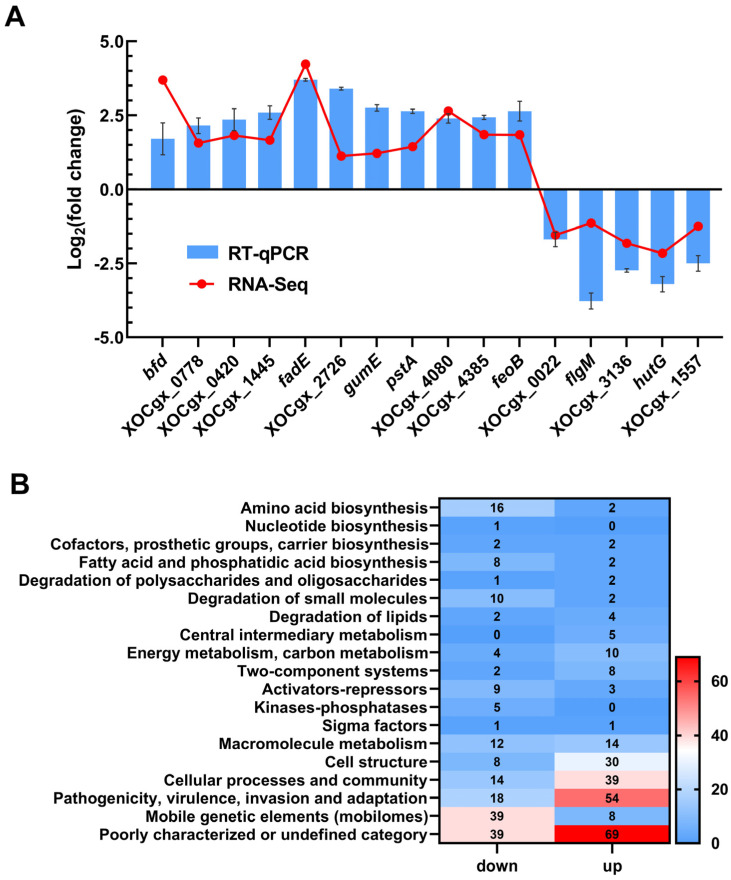
TfmR is a global regulatory protein that affects the expression of many genes. (**A**) A total of 16 genes from the transcriptome data were selected to verify the accuracy of the transcriptome by RT–qPCR. Values are the means ± SD (*n* = 3 biological replicates). (**B**) Functional categories of differentially expressed genes (DEGs) (|log2(fold change)| ≥ 1) in Δ*tfmR* mutants. The transcriptomes of *Xoc* strains cultured in NB medium were investigated by RNA–Seq. In the 446 DEGs of Δ*tfmR* mutants, 191 genes were significantly down–regulated and 255 genes significantly up–regulated.

Analysis of the transcriptome data revealed differential expression of 446 genes in the mutant Δ*tfmR*, with 191 genes significantly down-regulated and 255 genes significantly up-regulated (Table S1). To validate the transcriptome data, we randomly selected 16 genes and assessed their relative expression levels using reverse transcription quantitative real-time PCR (RT-qPCR). The results showed broadly consistent expression patterns with those observed in the transcriptome analysis (Figure 2A).

Functional clustering analysis of the differentially expressed genes, based on the genome annotation of *Xoc* strain GX01, revealed that they could be categorized into 19 functional categories. Among these, 108 genes were classified as belonging to the ‘poorly characterized or undefined category’. The majority of the remaining genes were associated with ‘mobile genetic elements (mobilomes)’, ‘pathogenicity, virulence, invasion and adaptation’, ‘cell structure’, ‘cellular processes and community’, ‘degradation of small molecules’, ‘amino acid biosynthesis’, and ‘fatty acid and phosphatidic acid biosynthesis’. Notably, 72 genes were categorized under ‘pathogenicity, virulence, invasion and adaptation’ (Figure 2B). These findings provide valuable insights into the regulatory mechanisms orchestrated by TfmR in *Xoc*.

### 2.3. Xoc TfmR Directly Binds to the Promoter of rpfG and Activates Its Transcription

**Figure 3:**
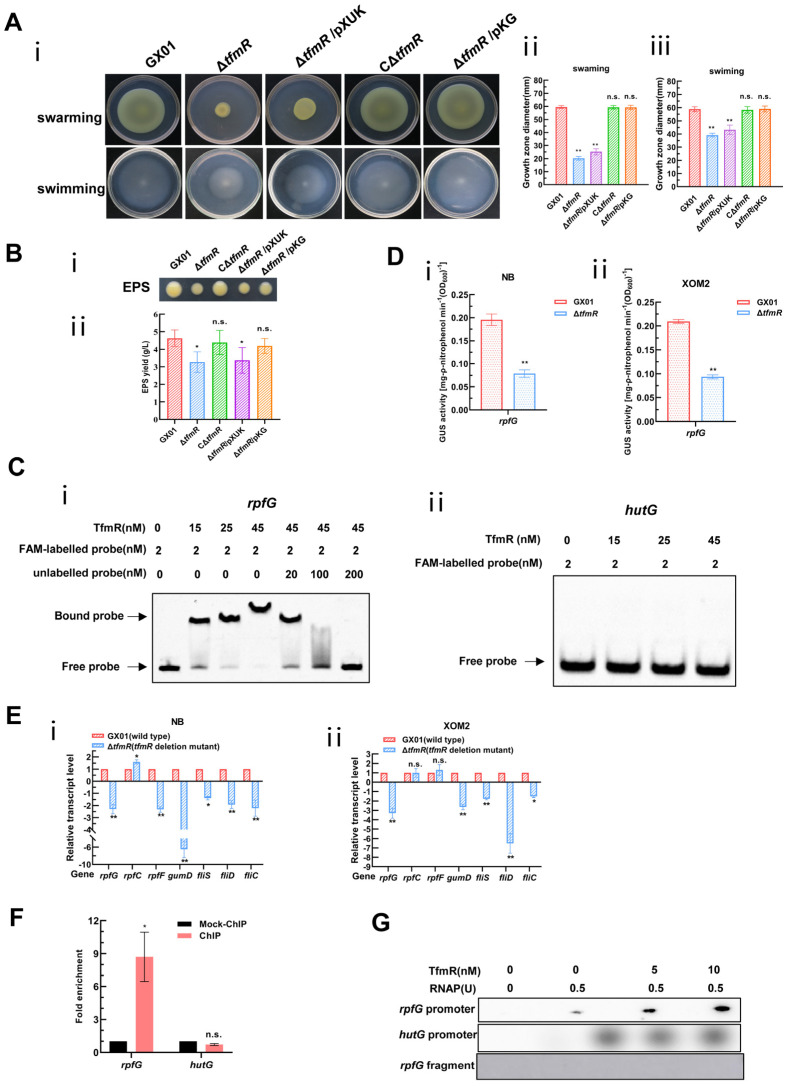
Constitutive expression of *rpfG* restores motility and EPS production of the mutant Δ*tfmR*. And *Xoc* TfmR binds directly to the promoter of RpfG and activates its transcription. (**A**) Constitutive expression of *rpfG* restores motility of the mutant Δ*tfmR*. (**i**) Example photo of a bacterial strain. (**ii**) Mean measurements of colony diameter for each strain on “swarming” plates. (**iii**) Mean measurements of colony diameter for each strain on “swimming” plates. Data shown are the mean ± SD (*n* = 10). Significance was determined by ANOVA and Dunnett’s post hoc test for comparison with to the wild type. ** *p* < 0.01; n.s., not significant. (**B**) Constitutive expression of *rpfG* restores the yield of the mutant Δ*tfmR* EPS. (**i**) *Xoc* strains were grown on NA plates supplemented with 2% sucrose for 3 days. (**ii**) *Xoc* strains were cultured in NB medium supplemented with 2% sucrose for 3 days and EPS was precipitated from the culture supernatant. Values given are the means ± SD of triplicate measurements from a representative experiment, and significance was determined by analysis of variance (ANOVA) and Dunnett’s post hoc test for comparison with the wild type. * *p* < 0.05; n.s., not significant. Similar results were obtained in two other independent experiments. (**C**) Electrophoretic mobility shift and competition assays of TfmR with the promoter region of *rpfG* (**i**) and *hutG* (**ii**) (negative control); the bound– and free–DNA fragments are marked with the words Bound probe and Free probe, respectively, and the concentrations are indicated at the top of each lane. (**D**) ß–Glucuronidase (GUS) activity of the *gusA* reporter of the *rpfG* gene promoter in the Δ*tfmR* mutant and the wild type in NB medium (**i**), or in XOM2 medium (**ii**). The data shown are the mean and standard deviation of three measurements. The experiment was repeated three times and similar results were obtained. Differences were evaluated by Student’s *t*-test (** *p* < 0.01; * *p* < 0.05; n.s., no significance at *p* ≤ 0.05). (**E**) Detection of Δ*tfmR* mutant and wild–type expression of *rpf* genes in NB medium (**i**), or XOM2 medium (**ii**), revealed by RT–qPCR analysis. Values are the means ± SD (*n* = 3 biological replicates). Differences were evaluated by Student’s *t*-test (** *p* < 0.01; * *p* < 0.05; n.s., no significance at *p* ≤ 0.05). (**F**) Fold enrichment of the promoter region of *rpfG* in the GX01/TfmR::3 × Flag–ChIP samples compared with the Mock–ChIP samples (with anti–HA antibody), as measured by ChIP–qPCR using *hutG* as the negative control. Data are presented as means ± SD (*n* = 3). Differences were evaluated using Student’s *t*-test (* *p* < 0.05; n.s., no significance at *p* ≤ 0.05). (**G**) In vitro transcription experiments showing TfmR activates the transcription of *rpfG*. RNA was produced from a 323 bp template DNA fragment containing the *rpfG* promoter using *E. coli* RNA polymerase (RNAP) holoenzyme. A 334 bp template DNA fragment containing the *hutG* promoter and a 150 bp template DNA fragment of the *rpfG* coding sequence were used as controls. Lane 1, template DNA alone; lane 2, template DNA with RANP; lanes 3–4, template DNA with RANP and 5 and 10 nM TrxA–TfmR.

Although our transcriptomic data showed that 191 genes were significantly downregulated in the mutant Δ*tfmR*, there were few genes with respect to pathogenicity among these, since the mutant Δ*tfmR* has a significant decrease in pathogenicity compared to the wild type, and the production of extracellular polysaccharides as well as motility is reduced (Figure 1A, Figure S3A and Figure 3A,B). This is very similar to the phenotype after deletion of the *rpf* gene in *Xanthomonas* [6,7,27,28,29,30]. We guessed that TfmR is likely to regulate the expression of the *rpf* gene. Therefore, we performed an electrophoretic mobility shift assay (EMSA) on the promoter regions of *rpfF*, *rpfC* and *rpfG*, and found that TfmR did bind to the promoter region of *rpfG* (Figure 3C and Figure S6A), but not to the promoter regions of *rpfF* and *rpfC* (Figure S5). After that, we investigated in depth the regulatory mechanism of TfmR on RpfG.

To further investigate, we engineered a reporter plasmid for RpfG, where its promoter sequence was fused with the *gusA* gene, enhancing *gusA* activity. The introduction of this reporter plasmid into both the wild-type and mutant-Δ*tfmR* strains, followed by cultivation in nutrient-rich (NB) and plant-mimicking (XOM2) media, revealed significantly lower β-glucuronidase (GUS) activity in the mutant Δ*tfmR* compared to the wild type in both conditions (Figure 3D).

Furthermore, analysis of *rpfG*, *rpfC*, and *rpfF* transcript levels via reverse transcription quantitative real-time PCR (RT-qPCR) corroborated diminished *rpfG* expression in the mutant Δ*tfmR* across both media types. Hence, we posit that TfmR activates *rpfG* expression in *Xoc*. Consistent with previous findings [6,30], reduced expression of genes *gumD*, *fliS*, *fliD*, and *fliC* was observed in the deletion mutant of *tfmR*, akin to the effects of *rpfG* deletion mutants, possibly due to decreased *rpfG* expression (Figure 3E).

Based on our previous EMSA experiments using TrxA-tagged TfmR proteins, it was indeed shown that there are mobility shifts in the DNA probes spanning the *rpfG* promoter region in vitro, which intensified with increasing TrxA-TfmR protein concentration. Notably, no shifted bands were observed in the presence of TrxA protein alone or with a control DNA fragment (Figure 3C and Figure S6A). Chromatin immunoprecipitation-qPCR (ChIP-qPCR) analysis further confirmed direct binding of TfmR to the *rpfG* promoter in vivo (Figure 3F and Figure S7), while in vitro transcription experiments demonstrated that TfmR induced *rpfG* transcription in the presence of RNA polymerase allosteric enzyme (RNAP) (Figure 3G). Collectively, these findings strongly support the hypothesis that TfmR regulates *rpfG* expression by directly binding to its promoter region, thus elucidating a crucial regulatory pathway in *Xoc* pathogenesis.

### 2.4. Constitutive Expression of rpfG Restores Mutant ΔtfmR Motility, EPS Production, Pathogenicity, and In Vivo Plant Growth

The significantly reduced pathogenicity of the *Xoc* GX01 mutant strain Δ*tfmR* implies that TfmR may be involved in other cellular processes required for full pathogenicity of *Xoc*, such as cell motility, and extracellular polysaccharide (EPS) production. To confirm that the effect of TfmR on these cellular processes is due to the regulation of *rpfG* expression, we constitutively expressed RpfG in the TfmR-deficient mutant Δ*tfmR* and determined its phenotype. To accomplish this, we utilized the pXUK plasmid to construct the cross-complementary strain Δ*tfmR*/pKG (Table S2), while we introduced the pXUK null plasmid into the mutant strain Δ*tfmR*, named Δ*tfmR*/pXUK (Table S2). The results showed that both the swarming and swimming abilities of the mutant strain Δ*tfmR* were significantly reduced, relative to that of the wild-type strain GX01 under the tested conditions, and that the complementary strain CΔ*tfmR* could completely restore the motility of mutant strain Δ*tfmR* (Figure 3A). Whereas the Δ*tfmR*/pXUK strain had similar motility to the mutant Δ*tfmR*, the Δ*tfmR*/pKG strain had similar motility to GX01 and the complementary strain CΔ*tfmR* (Figure 3A). Under the tested conditions, the yield of EPS of the deletion-mutant Δ*tfmR* was significantly lower than that of the wild-type strain GX01, and the complementary strain CΔ*tfmR* could completely restore its phenotype; the yield of EPS of the Δ*tfmR*/pXUK strain was similar to that of Δ*tfmR*, and the yield of EPS of the Δ*tfmR*/pKG strain was also similar to that of GX01 and CΔ*tfmR* (Figure 3B). The above results suggest that constitutive expression of *rpfG* can restore motility and EPS production in Δ*tfmR* strains.

**Figure 4:**
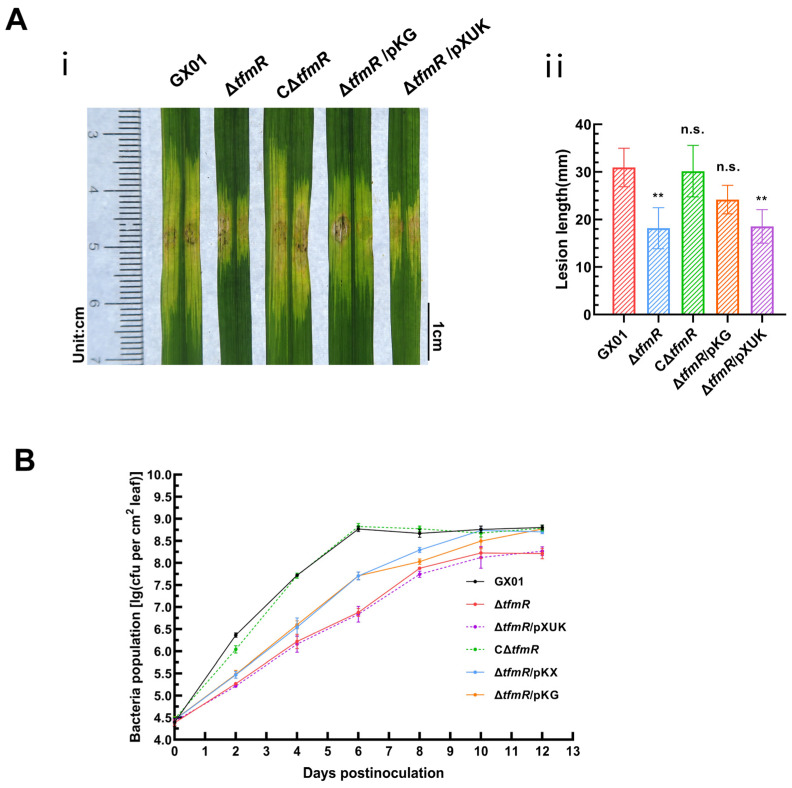
Constitutive expression of *rpfG* restores restores Δ*tfmR* mutant pathogenicity and proliferation in host plants. (**A**) Constitutive expression of *rpfG* can partially characterize the pathogenicity of the mutant Δ*tfmR*. (**i**) Phenotypic chart of lesion length. (**ii**) Statistical chart of lesion length, values are means ± SD (*n* = 30). Significance was determined by ANOVA and Dunnett’s post hoc test to compare with the wild type. ** *p* < 0.01; n.s., not significant. The experiment was repeated three times, with similar results. (**B**) Both constitutive expression of *hrpX* and constitutive expression of *rpfG* partially restored the proliferative capacity of the mutant Δ*tfmR* in the host rice plant. Data are shown as the mean ± SD (*n* = 3 biological repeats).

To test the significant reduction in pathogenicity of the mutant strain Δ*tfmR* in relation to RpfG, we inoculated the wild-type strain GX01, mutant strain Δ*tfmR*, complementary strain CΔ*tfmR*, constitutively expressed strain Δ*tfmR*/pKG and Δ*tfmR*/pXUK strain, respectively, into the host rice plant (*Oryza sativa* L. ssp. *Japonica* cultivar *Nipponbare*) by the leaf-infiltrating method. The results showed that the length of diseased spots was significantly lower than that of the wild-type GX01 in both the mutant strain Δ*tfmR* and the Δ*tfmR*/pKG strains; both had similar spot lengths that were significantly lower than that of the wild-type GX01, whereas the complementary strain CΔ*tfmR* could fully recover to wild-type levels and the Δ*tfmR*/pKG strain produced spots that could mostly recover to wild-type levels (Figure 4A and Figure S3B). In our subsequent experiments to detect the proliferation of the pathogen in host tissues, we could observe that the ability of the Δ*tfmR*/pKG strain to proliferate in the host plant was also mostly restored to that of the wild type (Figure 4B). These results suggest that constitutive expression of *rpfG* can largely restore the pathogenicity and growth of the Δ*tfmR* strain in host plants.

### 2.5. Xoc TfmR Directly Binds to the Promoter of hrpX and Activates Its Transcription

According to a previous report by Sun et al., RpfG negatively regulates the expression of *hrpG* and *hrpX* genes [6]. Given that TfmR positively regulates RpfG expression, we constructed two GUS reporter plasmids for two key *hrp*-regulated genes, *hrpG* and *hrpX*, as per the previous method, and introduced them into both the wild-type and mutant-Δ*tfmR* strains.

**Figure 5:**
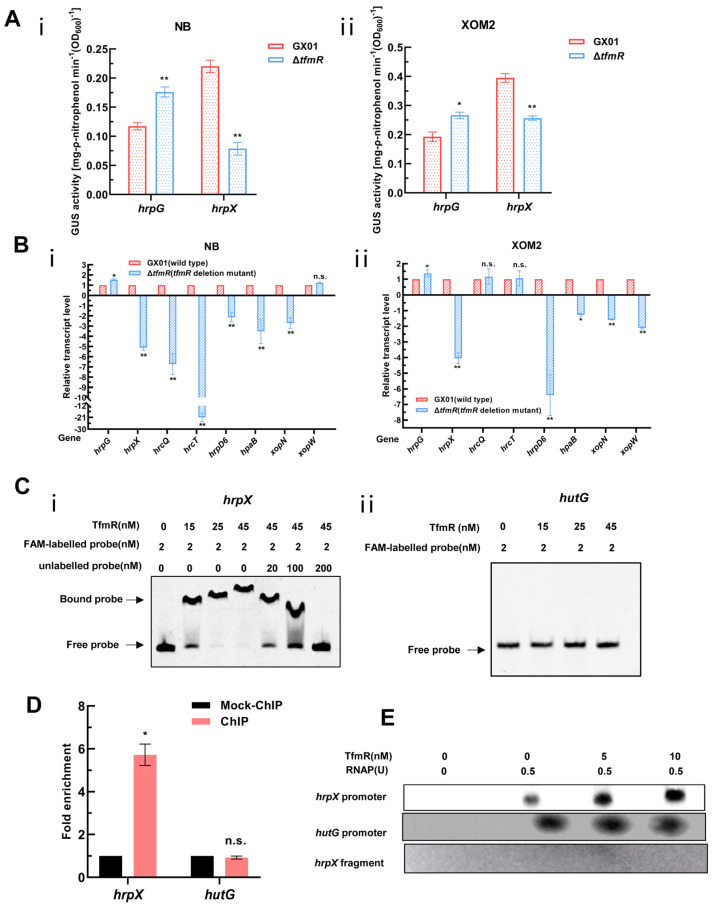
*Xoc* TfmR binds directly to the promoter of HrpX and activates its transcription. (**A**) ß–Glucuronidase (GUS) activity of the *gusA* reporter of the *hrpG* and *hrpX* gene promoter in the Δ*tfmR* mutant and the wild type in NB medium (**i**), or in XOM2 medium (**ii**). The data shown are the mean and standard deviation of three measurements. The experiment was repeated three times and similar results were obtained. Differences were evaluated by Student’s *t*-test (** *p* < 0.01; * *p* < 0.05; n.s., no significance at *p* ≤ 0.05). (**B**) Detection of Δ*tfmR*–mutant and wild-type expression of T3SS genes in NB medium (**i**), or XOM2 medium (**ii**) revealed by RT–qPCR analysis. Values are the means ± SD (*n* = 3 biological replicates). Differences were evaluated by Student’s *t*-test (** *p* < 0.01; * *p* < 0.05; n.s., no significance at *p* ≤ 0.05). (**C**) Electrophoretic mobility shift and competition assays of TfmR with the promoter region of *hrpX* (**i**) and *hutG* (**ii**) (negative control); the bound– and free–DNA fragments are marked with the words Bound probe and Free probe, respectively, and the concentrations are indicated at the top of each lane. (**D**) Fold enrichment of the promoter region of *hrpX* in the GX01/TfmR::3 × Flag–ChIP samples compared with the Mock–ChIP samples (with anti–HA antibody), as measured by ChIP–qPCR using *hutG* as the negative control. Data are presented as means ± SD (*n* = 3). Differences were evaluated using Student’s *t*–test (* *p* < 0.05; n.s., no significance at *p* ≤ 0.05). (**E**) In vitro transcription experiments showing TfmR activates the transcription of *hrpX*. RNA was produced from a 371 bp template DNA fragment containing the *hrpX* promoter using *E. coli* RNA polymerase (RNAP) holoenzyme. A 334 bp template DNA fragment containing the *hutG* promoter and a 161 bp template DNA fragment of the *hrpX* coding sequence were used as controls. Lane 1, template DNA alone; lane 2, template DNA with RANP; lanes 3–4, template DNA with RANP and 5 and 10 nM TrxA–TfmR.

As anticipated, the GUS enzyme activity of the HrpG reporter in the mutant Δ*tfmR* was significantly higher than that of the wild type in both NB and XOM2 medium (Figure 5A). Interestingly, however, the GUS enzyme activity of the HrpX reporter in mutant Δ*tfmR* was significantly lower than that of the wild type in both media (Figure 5A). To validate this result, we conducted RT-qPCR to analyze the expression changes of a set of T3SS or T3SS-related genes in both the wild type and mutant Δ*tfmR*. The results revealed that while the expression of *hrpG* in both media of mutant Δ*tfmR* was significantly higher than that of the wild type, the expression of *hrpX* and most T3SS or T3SS-related genes in mutant Δ*tfmR* was significantly lower than that of the wild type in both the NB and XOM2 medium (Figure 5B). These findings are consistent with the results of the GUS reporter system, suggesting a significant decrease in HrpX expression in the mutant Δ*tfmR*, potentially indicating direct regulation of HrpX expression by TfmR.

To investigate whether TfmR directly binds to the HrpX promoter to activate its expression, we conducted EMSA experiments in vitro. The results demonstrated that TrxA-TfmR protein could bind to the promoter region of HrpX under experimental conditions, while TrxA protein did not exhibit binding under the same conditions (Figure 5C and Figure S6B). Notably, under the same conditions, the TrxA-TfmR protein also failed to bind to the promoter region of HutG (negative control) (Figure 5C). Subsequently, ChIP-qPCR experiments conducted in vivo in bacteria revealed significant enrichment of the HrpX promoter region in the TfmR-ChIP samples compared to mock ChIP control samples, confirming the direct binding of TfmR to the HrpX promoter region in vivo. Conversely, no binding was detected in the negative control *hutG* promoter region (Figure 5D and Figure S7). These experiments conclusively demonstrated that TfmR directly binds to the promoter of HrpX.

Following this, we conducted in vitro transcription experiments, which showed that the transcription products of *hrpX* increased with the concentration of TfmR protein, while the transcription products of *hutG* remained unchanged (Figure 5E). In summary, these experiments confirm that TfmR directly binds to the promoter of HrpX and activates its transcription.

### 2.6. Constitutive Expression of hrpX Restores ΔtfmR Mutant HR, Pathogenicity, and Proliferation in Host Plants

**Figure 6:**
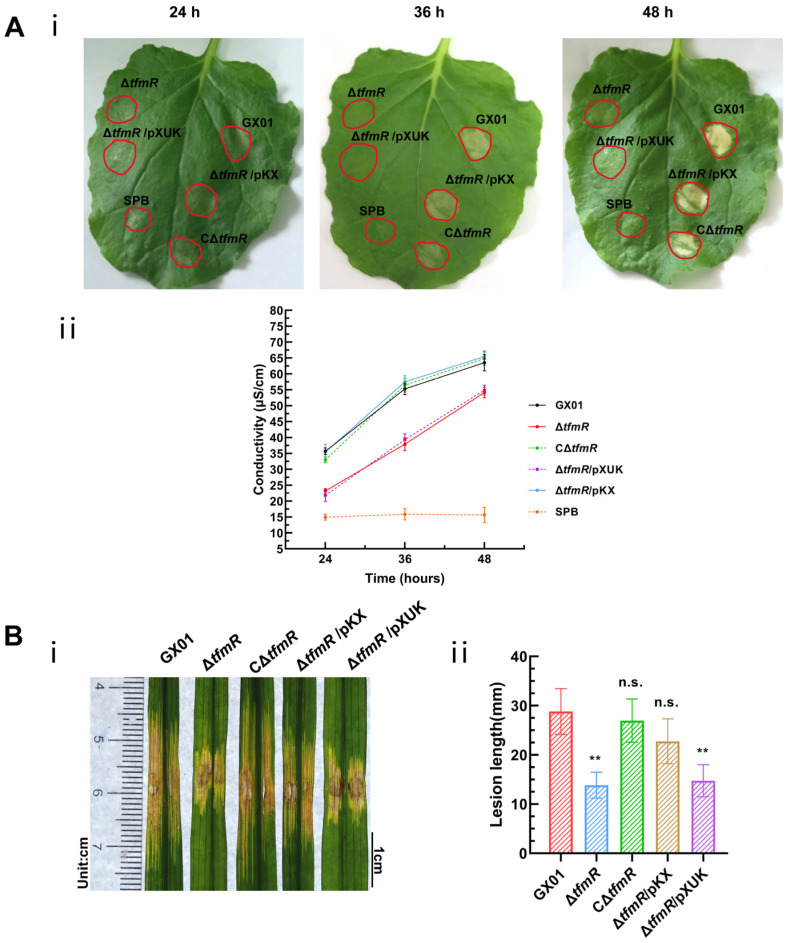
Constitutive expression of *hrpX* restores Δ*tfmR* mutant HR and pathogenicity. (**A**) Constitutively expressing *hrpX* in the Δ*tfmR* mutant restores its ability for hypersensitive response (HR) induction in nonhost plant *N. benthamiana*. (**i**) The HR symptoms recorded at 24, 36, and 48 hours post-inoculation (hpi). Three replications were performed in each experiment, and the experiment was repeated three times. The results presented are from a representative experiment, and similar results were obtained in all other independent experiments. (**ii**) The electrolyte leakage from *N. benthamiana* leaves inoculated with *Xoc* strains. Three samples were taken for each measurement in each experiment. Data are shown as mean and standard deviation. (**B**) Constitutive expression of *hrpX* partially restores the pathogenicity of mutant Δ*tfmR*. (**i**) Phenotypic chart of lesion length. (**ii**) Statistical chart of lesion length, values are means ± SD (*n* = 30). Significance was determined by ANOVA and Dunnett’s post hoc test to compare with the wild type. ** *p* < 0.01; n.s., not significant. The experiment was repeated three times with similar results.

The impact of TfmR mutation on the expression of the T3SS gene prompted an evaluation of its effect on HR induction using an osmotic assay. While the wild-type strain and the complementary strain CΔ*tfmR* exhibited pronounced HR symptoms at 24 h, the mutant strain Δ*tfmR* showed observable HR symptoms only at 36 h, indicating delayed and attenuated HR (Figure 6A). To confirm that TfmR’s effect on plant T3SS is mediated by the modulation of *hrpX* expression, we tested a TfmR mutant constitutively expressing *hrpX* in various HR-induced assays.

A plasmid constitutively expressing *hrpX* was constructed and introduced into the mutant Δ*tfmR*, designated as Δ*tfmR*/pKX (Table S2). This strain exhibited HR comparable to the wild-type strain GX01. Quantitative assessment of HR induction using an electrolyte leakage assay revealed that both the mutant-Δ*tfmR* and Δ*tfmR*/pXUK strains had significantly lower HR than GX01 at 24 h, 36 h, and 48 h. Conversely, both the complementary strain CΔ*tfmR* and the constitutively expressed strain Δ*tfmR*/pKX showed HR levels similar to the wild-type strain (Figure 6A).

To examine the relationship between the decrease in pathogenicity of mutant Δ*tfmR* and the T3SS gene, pathogenicity tests were conducted. Results mirrored those of the HR experiments, with both mutant strains Δ*tfmR* and Δ*tfmR*/pXUK exhibiting significantly reduced pathogenicity compared to GX01. However, the complementary strain CΔ*tfmR* fully restored pathogenicity, while the constitutively expressed strain Δ*tfmR*/pKX partially restored it (Figure 6B and Figure S3C). Additionally, the in-plant growth curve demonstrated that the mutant strain Δ*tfmR* exhibited significantly weaker growth, while the constitutively expressed strain Δ*tfmR*/pKX partially restored proliferation in plants (Figure 4B).

These results collectively indicate that constitutive expression of *hrpX* can restore HR, pathogenicity, and proliferation of the Δ*tfmR* strain in plants, suggesting that TfmR regulates the T3SS gene by modulating *hrpX* expression.

## 3. Discussion

**Figure 7:**
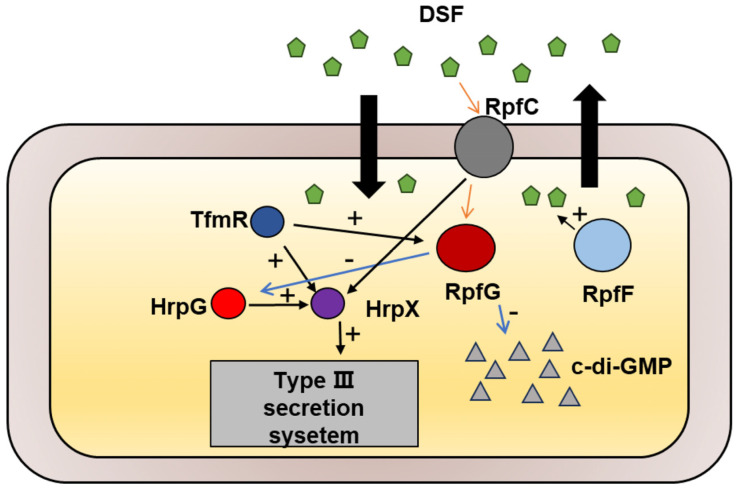
Schematic representation of the genetic regulation between TfmR and its target genes in *Xoc*. TfmR is involved in activating the expression of RpfG and HrpX.

The TetR family of transcriptional regulators governs various bacterial processes [22], and our study has identified TfmR as a significant regulator in the *Xoc* GX01 strain. Through our investigations, we have demonstrated that *Xoc* TfmR acts as a global regulator, influencing diverse cellular processes including motility, extracellular enzymes, EPS production, bacterial proliferation, and T3SS gene expression. Specifically, we have identified TfmR as a key regulator of two pathogenesis-related pathways in *Xoc*. Deletion of TfmR resulted in reduced expression of *rpfG* and T3SS genes. Our findings indicate that TfmR directly promotes the expression of *rpfG* and *hrpX* genes, underscoring its crucial role in positively regulating pathogenicity in *Xoc* (Figure 7).

The *rpf* genes cluster in the *Xanthomonas* spp. plays a pivotal role in pathogenicity, community sensing, and various cellular processes [7]. In the *Xanthomonas* spp., the two-component system comprising RpfG and the sensor kinase RpfC responds to the cell–cell signaling molecule DSF to regulate the synthesis of virulence factors [7,11,29]. RpfG, with its HD-GYP cyclic di-GMP phosphodiesterase domain, is a well-studied regulator within the *rpf* gene cluster. Studies across different *Xanthomonas* species have highlighted its importance in pathogenicity, motility, and virulence-factor production [6,7,27,28,31,32,33,34,35]. RpfG was critical for pathogenicity and colonization of rice in the *Xoc* strain BLS256, and deletion of the *rpfG* resulted in a significant decrease in the yield of EPS in the mutant [6]. In *Xanthomonas oryzae* pv. *oryzae* (*Xoo*), RpfG was positively correlated with pathogenicity and proliferation in the host plant, but did not affect *Xoo* growth in enriched medium [31,32]. In *Xanthomonas campestris* pv. *campestris* (*Xcc*), knockout of *rpfG* resulted in a significant reduction in pathogenicity, reduced cellulase, reduced EPS production, and reduced motility [7,27,28]. In *Xanthomonas albilineans* (*Xal*), single deletion of the *rpfG* and *rpfC* genes resulted in mutants with no change in virulence or swimming ability, but double deletion of the *rpfG* and *rpfC* genes resulted in a slight decrease in virulence and severely impaired swimming ability [35]. Since the contribution of the *rpfG* gene to pathogenicity in *Xanthomonas* is particularly important, a number of genes have also been identified that can regulate the expression of the *rpfG* gene. In the *Xoc* RS105 strain, deletion of the *zwf* gene leads to an increase in RpfG at the transcriptional level [34]. The expression of two genes, *rpfC* and *rpfG*, was down-regulated in *Xoo* with the deletion of the *thiG* gene [33]. Importantly, to the best of our knowledge, no transcriptional regulator that can directly regulate RpfG in *Xanthomonas* has been reported yet. Our experimental results for EMSA and ChIP-qPCR indicate that TfmR can directly bind the promoter region of RpfG (Figure 3). And, according to our in vitro transcription, RT-qPCR and GUS enzyme activity experiments, TfmR is a positive transcriptional regulator for the *rpfG* gene in the *Xoc* GX01 strain (Figure 3). However, the regulation of the *rpfG* gene is very complex. Therefore, the next direction of research should focus on the regulatory network of regulatory factors for the *rpfG* gene.

Moreover, our findings regarding TfmR’s regulation of *hrpX* expression elucidate a previously unexplored aspect of *Xoc* pathogenesis. T3SS is essential for Gram-negative bacterial pathogens [12,13]. In *Xanthomonas*, virulence is dependent on the secretion and transport of T3SS effectors [12]. These effectors are regulated by two master transcription regulators, HrpG and HrpX [17]. Great efforts have been made to reveal the regulatory network of the HrpG/HrpX regulon [17]. The GntR-family transcriptional regulators HpaR1/YtrA from *Xcc* and *Xcci* negatively regulate *hrpG* expression in plant minimal medium, and positively regulate gene expression in plants [36,37]. In *Xcc*, HpaS is a membrane-bound histidine kinase sensor that forms a two-component signal transduction system with HrpG and activates HrpG activity by phosphorylation [38]. In the *Xoc* RS105 strain, there is a cross-talk between CheA/VemR and HpaS/HrpG-mediated signaling events that coordinate the *hrp* gene expression [39]. In addition to HrpG, regulatory factors have been identified in other *Xanthomonas* spp. that can directly regulate HrpX. In *Xoo*, the Sigma factor 70 RpoD directly binds to the promoters of *hrpG* and *hrpX* to activate T3SS gene expression [40]. And the GntR-family transcriptional regulator Sar can positively regulate the expression of the T3SS gene by directing the promoter of *hrpG* and *hrpX* in *Xoo* [20]. In *Xcc*, the *fis*-type transcriptional regulator Flp interacts with the *hrpX* promoter and positively regulates its expression [41]. It is noteworthy that no proteins (except for HrpG) have been reported in *Xoc* that directly positively regulate the HrpX. Through EMSA, ChIP-qPCR, and in vitro transcription experiments, we have confirmed in the *Xoc* GX01 strain TfmR’s direct binding to the *hrpX* promoter, thereby activating its transcription (Figure 5). Although in previous studies TfmR could indirectly regulate the expression of T3SS genes in *Xcci 306* by regulating fatty acid metabolism, TfmR could not directly bind to the promoters of *hrpX* and *hrpG* in the *Xcci* 306 strain [22]. Further studies are needed regarding the differences between the two proteins, *Xoc* TfmR and *Xcci* TfmR, in regulating T3SS gene expression.

In addition, differences in culture environments lead to changes in the expression of *hrpG/hrpX* [17]. According to previous studies, in *Xanthomonas*, *hrpG/hrpX* gene expression was repressed in nutrient-rich medium but induced in plants and minimal medium [16,17]. Although many proteins regulating *hrpG/hrpX* have been identified, the exact mechanism is currently unknown. We performed the transcriptome analysis in nutrient-rich medium, which may be the reason why we did not find significant changes in *hrpX* expression in our transcriptome. According to Sun et al., in *Xoc*, RpfG negatively regulates *hrpG* expression [6], whereas TfmR positively regulates RpfG expression. The slightly higher expression of *hrpG* in the mutant compared to the wild type in minimal culture is likely to be caused by the decreased expression of RpfG. Our results demonstrate that TfmR positively regulates *hrpX* and T3SS gene expression while concurrently activating *rpfG* gene expression. This discovery underscores the intricate regulatory mechanisms governing *Xoc* virulence.

In conclusion, our work highlights the multifaceted signaling pathways underlying *Xoc* virulence and describes TfmR as a pivotal regulator orchestrating T3SS gene expression, HR induction, and pathogenesis. By elucidating the regulatory mechanisms involving TfmR, RpfG, and HrpX, we provide valuable insights for developing strategies to combat plant pathogens such as *Xoc*.

## 4. Materials and Methods

### 4.1. Bacterial Strains, Plasmids, and Growth Conditions

The bacterial strains and plasmids used in this work are enumerated in Table S2. *E. coli* (*Escherichia coli*) strains were cultured in LB (Luria Bertani) medium (containing 5 g yeast extract, 10 g NaCl, and 10 g tryptone per liter) or on LB agar plates (LB supplemented with 30 g agar per liter) at 37 °C. *Xoc* strains were cultured in NB medium (comprising 1 g yeast extract, 3 g beef extract, 5 g polypeptone, and 10 g sucrose per liter), NA (NB with 30 g agar per liter), or the minimal medium XOM2 [42]. Antibiotics were used, according to the concentrations as required: kanamycin at 25 µg/mL, rifampicin at 50 µg/mL, ampicillin at 100 µg/mL, spectinomycin at 50 µg/mL and Tet at 5 µg/mL for *Xoc* strains and 15 µg/mL for *E. coli* strains.

### 4.2. Nucleic Acid Manipulations

The procedure for nucleic acid manipulation was conducted according to the method outlined by Sambrook et al. [43]. Conjugation between *Xoc* and *E. coli* strains was executed as detailed by Turner et al. [37]. The necessary enzymes—restriction endonuclease, T4 DNA ligase, and Pfu polymerase—were supplied by Promega (Shanghai, China). Total RNA was extracted from *Xoc* strain cultures utilizing the Total RNA Extraction Kit (Invitrogen, Carlsbad, CA, USA), while cDNA synthesis was carried out using the cDNA Synthesis Kit (Invitrogen). For RT-qPCR, the obtained cDNA was diluted and utilized as a template with selected primers targeting specific genes. RT-qPCR was performed in the qPCR thermocycler (Analytik jena qTOWER2.0, Jena, Germany). Each reaction comprised ChamQ universal SYBR qPCR master mix (Vazyme Nanjing, China), appropriate primers, and cDNA. The relative mRNA levels were normalized relative to those of the target genes in the wild-type strain GX01 (equal to 1). The expression levels of the 16S rRNA genes were used as an internal standard. Triplicate RT-qPCR assays were conducted, and all primer sequences applied in this study are listed in Table S3.

### 4.3. Construction of Mutant Strains

The deletion mutant in *Xoc* was constructed using the previously employed homology double-swap method. As an example, the mutant Δ*tfmR* (XOCgx_1556) was constructed as follows [4]. PCR was used to amplify 425 bp of the upstream sequence and 309 bp of the downstream sequence of the *tfmR* gene, utilizing the appropriate primers. The two fragments were fused with the suicide plasmid pK18mobsacB [44] and transformed into *E. coli* DH5α. The recombinant plasmid obtained was introduced into *Xoc* using triparental conjugation with the help of plasmid pRK2073 [45]. Screening for triparental conjugates was carried out using NA plates containing Rif with Kan antibiotics. The single colonies that could grow on the double-resistant plates were all those in which the recombinant plasmid was integrated into the *Xoc* genome by the first homologous exchange, called a single exchanger. Since the *sacB* gene in the pK18mobsacB plasmid is a sucrose-lethal gene, the high concentrations of sucrose are utilized to force a second homologous exchange of the single exchanger. This resulted in the deletion mutant Δ*tfmR*.

For complementation of the Δ*tfmR*, the full length (606 bp) of *tfmR* was amplified by PCR from the total DNA of the *Xoc* strain GX01 and inserted into the pXUK vector [26] (adapted from an endogenous plasmid in the *Xoc* strain GX01), creating the plasmid pKC*tfmR*. For constitutive expression of RpfG or HrpX in Δ*tfmR*, we inserted the full-length (1137 bp) of *rpfG* or the full length (1431 bp) of *hrpX* into the pXUK vector, named pKG or pKX, respectively. These plasmids, including the pXUK null vector, were introduced into the Δ*tfmR* by triparental mating, respectively.

*Xoc* strains chromosomally encoding the proteins fused with a 3 × Flag-tag at the C-terminus were constructed using the method previously described [4]. Using the genomic DNA of strain GX01 as a template and the corresponding primers, a 1069 bp DNA fragment consisting of a 425 bp sequence upstream of the *tfmR* start codon, a 606 bp coding sequence for *tfmR*, and a 38 bp coding sequence for Flag was obtained by PCR amplification. Meanwhile, PCR amplification using the corresponding primers resulted in a 362 bp DNA fragment comprising 50 bp of the Flag coding sequence, 3 bp of the stop codon of *tfmR* and 309 bp downstream of the *tfmR* stop codon. The two fragments were jointed using overlap extension PCR, and the resulting recombinant fragment was cloned into the suicide plasmid pK18mobsacB. The plasmid was further introduced into the *Xoc* strain GX01 by conjugation. The transconjugants were screened on selective agar plates and were confirmed by DNA sequencing. This constructed variant strain was named as GX01/TfmR::3 × Flag.

### 4.4. Pathogenicity Tests, HR Assays, Leakage Assays and in-Plant Growth Curve

To assess the pathogenicity of *Xoc* in the host rice plants (*Oryza sativa* L. ssp. *Japonica* cultivar *Nipponbare*) the infiltration method was employed, as previously outlined [4]. Rice seedlings were cultivated in the greenhouse for 6 weeks before being inoculated with *Xoc* strains on their leaves. The *Xoc* strains, collected from overnight cultures, were washed and adjusted to a consistent final density (OD_600_ of 0.3, approximately 1 × 10^8^ CFU/mL). These resuspended bacterial cells were inoculated into 6-week-old rice leaves under relevant conditions, and the resulting lesions and symptoms were evaluated 14 days post-inoculation.

HR was tested on *Nicotiana benthamiana* leaves, as previously described [5]. Following a similar procedure, *Xoc* cells from cultures were washed and resuspended in sodium phosphate buffer (SPB, 5.8 mM Na_2_HPO_4_ and 4.2 mM NaH_2_PO_4_, pH 7.0) to an OD_600_ of 0.5 (5 × 10^8^ CFU/mL). Resuspended bacterial cells were infiltrated into the *N. benthamian*a leaves. Symptoms were monitored, and conductivity measurements were taken at 24, 36, and 48 h post-inoculation. For conductivity measurements, samples (four 0.4 cm^2^ leaf disks) were collected using a punch. These obtained leaf discs were immersed in 10 mL of ultrapure water and shaken at 200 rpm for 30 min. Then the leaf disk was removed and the conductivity of the liquid was measured with a DDS-307A conductivity meter.

Quantification of bacterial growth in plants was carried out as per prior procedures [4]. To elaborate, the *Xoc* bacterial solution adjusted to a certain concentration was inoculated into the rice leaves, and one infiltrated leaf per group of inoculated plants was sampled and homogenized every 48 h. The homogenates were serially diluted with NB medium and then coated onto selective NA plates with appropriate antibiotics, and after a 3-day incubation period, colony-forming units (CFUs) were counted.

### 4.5. Stress Tolerance Assay

To investigate the susceptibility of the *Xoc* strain to various environmental stresses, such as heavy metal salts (CuSO_4_), sodium dodecyl sulfate (SDS), phenol, and hyperosmotic challenge (NaCl), the minimum inhibitory concentration (MIC) method was employed [4]. In brief, the *Xoc* strain was cultured overnight and subsequently diluted to an OD_600_ of 1, and the diluted cultures were further diluted and incubated onto corresponding NA plates supplemented with different reagents. After 3 days of incubation at 28 °C, the number of surviving colonies was counted.

### 4.6. Extracellular-Polysaccharide and -Enzyme Assays

Extracellular-polysaccharide (EPS) and extracellular-enzyme assays were conducted, following previously established protocols [4,5]. For EPS production analysis, the *Xoc* strain was spotted on NA plates supplemented with 2% sucrose and incubated for 3 days. To quantify EPS production, the *Xoc* strain was cultured in NB medium containing 2% sucrose at 28 °C with shaking at 220 rpm for 3 days. The EPS was then precipitated from the culture supernatant using anhydrous ethanol. 

To assess protease and amylase activities, *Xoc* strains were spotted on NA plates containing skim milk (for protease) or soluble starch (for amylase) and incubated for 2 days. For quantification of enzymes, bacterial cells were cultured in NB medium for 24 h and adjusted to the same concentration.

### 4.7. Motility Assay

The method for detecting cell motility followed the previously described protocol [4]. To begin, the *Xoc* strain culture that had been incubated overnight was rewashed and resuspended to achieve the same concentration (OD_600_ of 0.5). Subsequently, 3 µL of the resuspended bacterial suspension was stabbed into 0.28% agar plates containing 0.03% bacterial peptone and 0.03% yeast extract for swimming, or spotted onto NA plates consisting of 2% sucrose and 0.6% agar for swarming. After a resting incubation at 28 °C for 5 days, the diameter of the area occupied by the bacterial cells was measured.

### 4.8. GUS Activity Assays

The measurement of GUS activity in *Xoc* strains followed the previously described protocol [41]. Both wild-type and mutant strains carrying the reporter plasmid were cultured at 28 °C in the appropriate medium. Bacterial cells were then collected and resuspended in 375 μL 1 mM p-nitrophenyl-β-d-glucuronide extraction buffer (comprising 50 mM sodium dihydrogen phosphate, 0.1% Triton X-100, and 10 mM β-mercaptoethanol, pH 7.2), and incubated for 10 min at 37 °C. This was followed by incubation with 200 mL of 2.5 M 2-amino-2-methyl-1,3-propylene glycol to terminate the reaction. GUS activity assays were performed in triplicate.

### 4.9. Overexpression and Purification of TrxA-TfmR Protein

To obtain purified TrxA-TfmR protein, the *tfmR* gene was first cloned and introduced into the expression vector pET32a. Subsequently, the recombinant plasmid was transformed into *E. coli* strain BL21. The protein expression purification process followed a well-established method, involving induction of the recombinant strain with isopropyl β-D-thiogalactopyranoside (IPTG) and purification of the fusion protein using Ni-NTA resin (Qiagen, Alameda, CA, USA).

### 4.10. Electrophoretic Mobility Shift Assay (EMSA)

The EMSA procedure followed a previously described protocol [4]. Briefly, DNA fragments labeled with FAM at the 5′ terminal were amplified using the appropriate primers. The purified protein was then incubated with the FAM-tagged DNA fragment in binding buffer [20 mm Tris-HCl, 10 mm NaCl, 1 mm ethylenediminetetraacetic acid (EDTA) and 1 mm dithiothreitol, pH 8.0] containing 1 μg of sonicated salmon sperm DNA and 3 μg of bovine serum albumin, incubated at 28 °C for 30 min, Samples were then loaded onto a 6% polyacrylamide-Tris-borate-EDTA (TBE) gel, and visualized.

### 4.11. In Vitro Transcription Assays

The in vitro transcription assays were conducted following a previously established protocol [4]. First, the corresponding DNA fragments (including the promoter region and a portion of the coding region of the target gene) were amplified using the appropriate primers. The TrxA-TfmR protein and DNA fragments were incubated in transcription buffer at 28 °C for 30 min. Then, the NTP mixture (250 μM each of ATP, CTP, and GTP; 250 μM biotin 16-UTP) and 0.5 U of *E. coli* RNA polymerase holoenzyme (New England BioLabs, Ipswich, MA, USA) were added and the reaction was carried out for 1 h at 37 °C. Transcription products were analyzed by electrophoresis at the end of the reaction. The obtained transcripts were visualized using a fluorescence imager screen (GE AI600, Boston, MA, USA).

### 4.12. Chromatin Immunoprecipitation–Quantitative PCR (ChIP-qPCR)

We performed ChIP-qPCR experiments as previously described, with only minor modifications [4]. Briefly, GX01/TfmR::3 × Flag reporter strains were cultured in medium to the corresponding OD_600_ values, collected and crosslinked using formaldehyde for cross-linking. Then sonication was performed and, for each ChIP sample, 50 μL of Flag antibody (agarose-conjugated) was added to 3.5 mL of bacterial lysate and incubated at 4 °C overnight. Unbound-DNA fragments were washed and bound-DNA fragments and proteins were eluted with 0.25 M glycine (pH 2.5). RNA was removed from the DNA samples by incubation with RNaseA for 2 h at 37 °C, and then proteins were removed by incubation with proteinase K for 2 h at 55 °C. The DNA samples were subsequently purified using a PCR purification kit (Qiagen, Alameda, CA, USA). To measure the enrichment of RpfG, HrpX, and HutG (negative control) in the ChIP DNA samples, relative-abundance quantitative PCR (qPCR) was performed, using the ChamQ Universal SYBR-qPCR Master Mix (Vazyme) and a real-time PCR thermocycler (Analytik jena qTOWER2.0; jena). The quantity of IP DNA (eluted DNA) was calculated as the percentage of the DNA present in the input DNA (10 μL sample taken prior to IP) using the ΔΔCt method to calculate the relative enrichment as the fold change [46]. The relative IP was calculated by normalizing IP to a mock ChIP using an anti-HA-tag monoclonal antibody (Solarbio, Beijing, China).

### 4.13. Transcriptome Analysis of the TfmR Mutant

Transcriptome analysis was conducted following the previously described protocol [5]. In brief, RNA was extracted from cell cultures with an OD_600_ of 0.8. To eliminate any contamination from genomic DNA, RNase-free DNase I was used. After quantification and quality assessment, the total-RNA samples were sent to Novogene (Beijing, China) for library construction and strand-specific RNA sequencing. The resulting clean reads were mapped to the genome of the *Xoc* strain GX01 (assembly ID 4726371), and gene expression levels were calculated using the RPKM (reads-per-kilobase per million-mapped-reads) method. Differentially expressed genes (DEGs) were identified based on a false discovery rate (FDR) of ≤0.05 and a |log2FC| (log2 of fold change) ≥ 1 threshold. The details of the DEGs can be found in Table S1.

### 4.14. Western Blotting

Western blotting was performed using the method previously used by Li et al. [47]. First, bacterial proteins separated by SDS-PAGE gels were electro-transferred onto polyvinylidene difluoride (PVDF) membranes (Millipore, Billerica, MA, USA). The membrane was then blocked with 1% milk for 1 h at room temperature, after which it was incubated with a 1:2500 dilution of anti-Flag-tagged mouse monoclonal antibody (Abmart) as the primary antibody, and then washed with Tris-buffer saline and Tween buffer (Tris 20 mM, NaCl 0.3 M, Tween 20 0.08%). Goat anti-mouse immunoglobulin G (IgG) (Beyotime Biotechnology) coupled with diluted 1:2500 horseradish peroxidase (HRP) was used as secondary antibody. Finally, the luminescent signal was detected according to the manufacturer’s instructions.

## Data Availability

The RNA sequencing data generated in this study are available in the NCBI SRA database under the accession codes PRJNA1098699. Other data are presented within the manuscript and Supplementary Materials.

## Supplementary Materials

The following supporting information can be downloaded at: https://www.mdpi.com/article/10.3390/ijms25115887/s1.
