# Supplementary material for: The Transcriptional Regulator TfmR Directly Regulates Two Pathogenic Pathways in Xanthomonas oryzae pv. oryzicola
Source: Int J Mol Sci. 2024 May 28;25(11):5887. doi: 10.3390/ijms25115887 (PMC11173191; doi:10.3390/ijms25115887)
Supplement: Supplementary file 1 [file ijms-25-05887-s001.zip › Supplementary Materials.pdf]

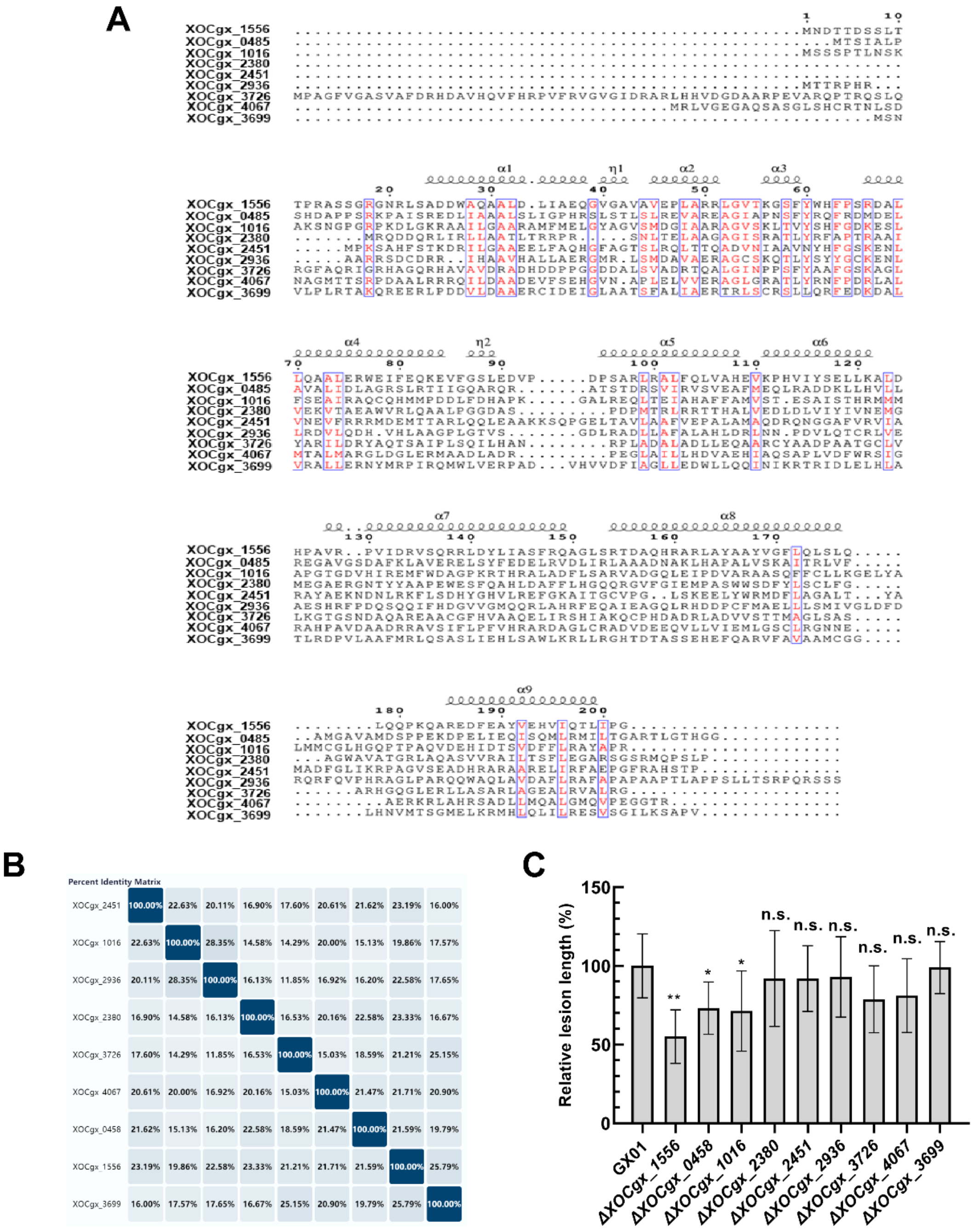

Figure S1. Sequence alignment, protein sequence identity analysis, and detection of pathogenicity of TetR-family transcriptional regulators in *Xoc* strain GX01. (A) Comparison of predicted amino acid sequences of all TetR family transcriptional regulators in *Xoc* GX01 strain, these protein sequences were taken from the NCBI website and compared using the ESPript 3.0 website. (B) Protein sequence identity analysis of all TetR family transcriptional regulators in *Xoc* GX01 strain. Percent identity matrix was generated using uniprot website using protein sequences from the NCBI website. (C) Pathogenicity assays for mutants of the TetR family of transcriptional regulators. A suspension of the mutant strain and the wild-type strain GX01 were inoculated into 6-week-old rice (*Oryza sativa* L. ssp. *Japonica* cultivar *Nipponbare*) using the leaf-infiltrating method, and lesion length was measured 14 days after inoculation. Values are means ± SD (n = 20). Significance was determined by ANOVA and Dunnett's post hoc test to compare with the wild type. *$P < 0.05$; **$P < 0.01$; n.s., not significant.

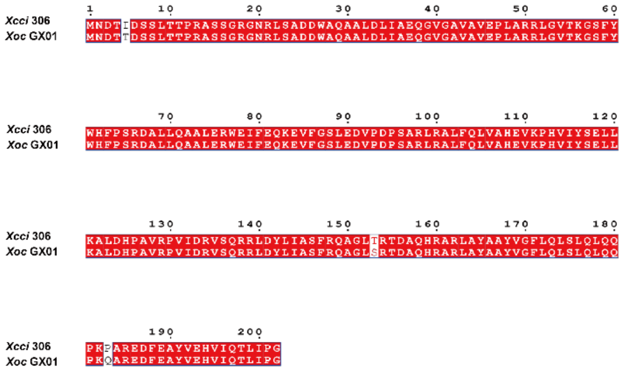

Figure. S2 Comparison of the predicted amino acid sequences of TfmR in *Xcci* strain 306 and TfmR in *Xoc* strain GX01. These protein sequences were obtained from the NCBI website and compared using the ESPript 3.0 website.

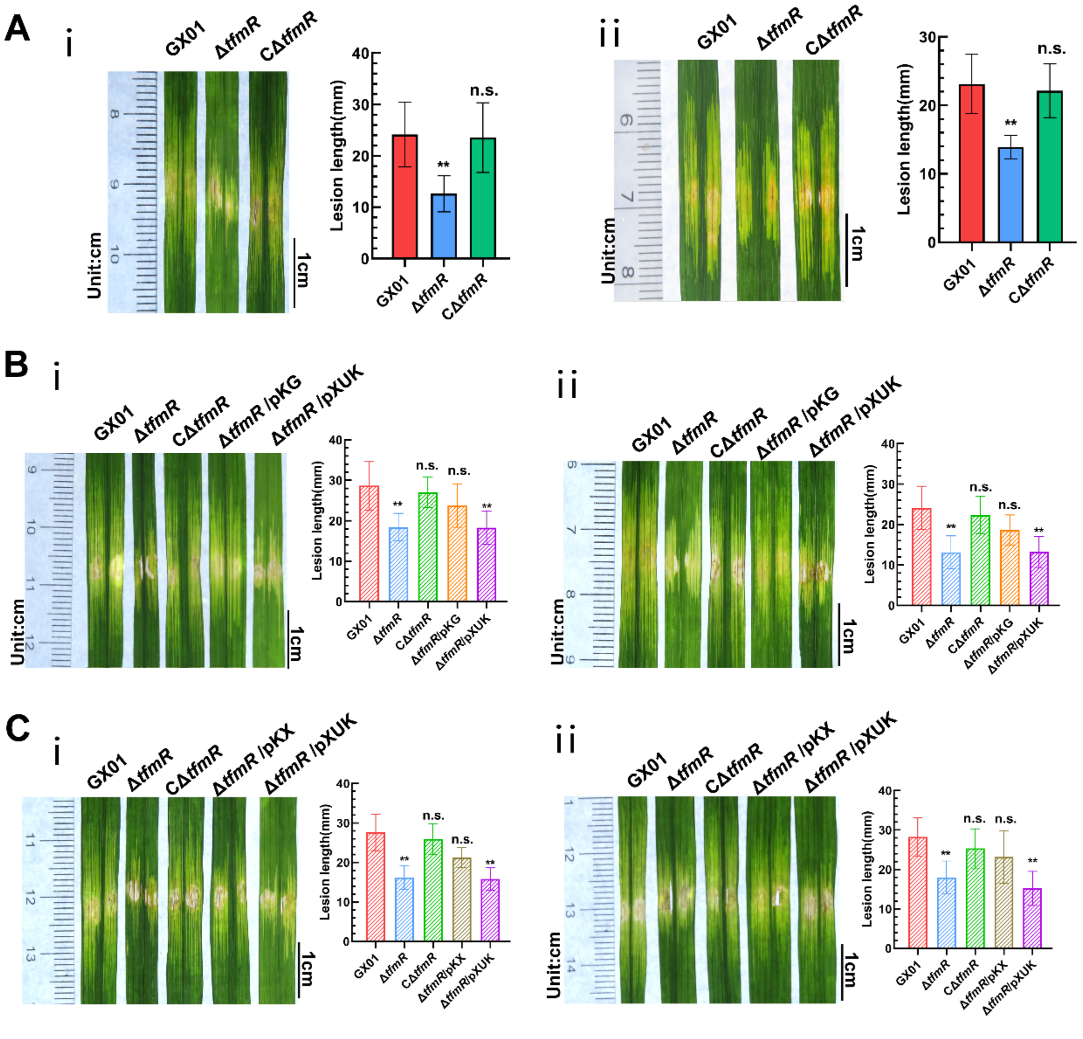

Figure. S3 *Xoc* strain pathogenicity assay. (A) Results of two replicate experiments for Figure 1A. (B) Results of two replicate experiments for Figure 4C. (C) Results of two replicate experiments for Figure 6B.

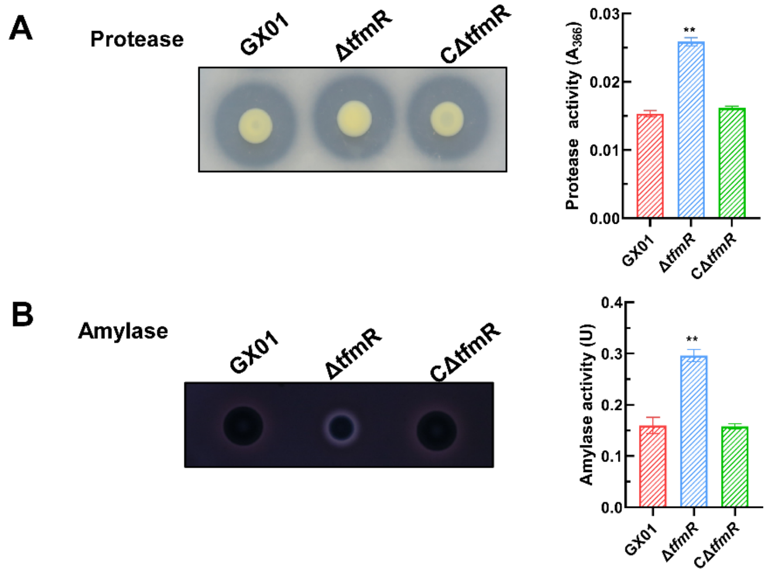

Figure. S4 Effect of the *tfmR* gene on extracellular enzymes. (A) Effect of the *tfmR* gene on the enzyme activity of extracellular proteases. (B) Effect of the *tfmR* gene on the enzyme activity of extracellular amylase. Data are the mean ± SD (n=3). Significance was determined by ANOVA and Dunnett's post hoc test for comparison to the wild type. **P < 0.01.

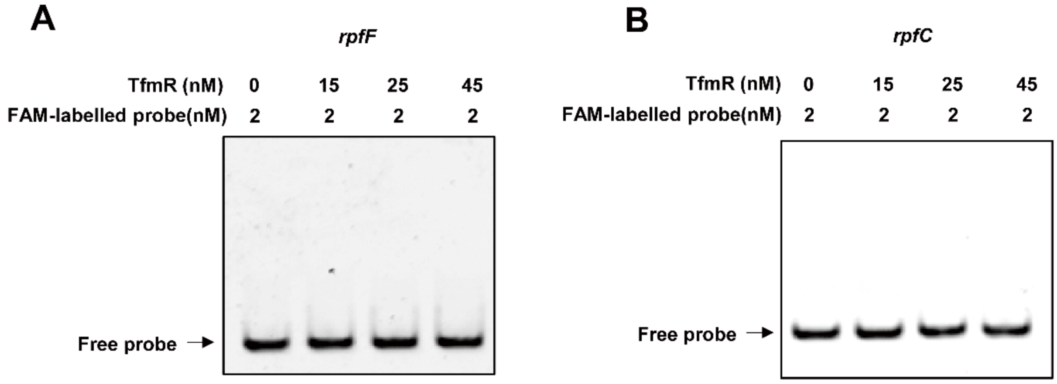

Figure. S5 TfmR do not bind the promoter regions of RpfF and RpfC. TfmR were subjected to EMSA with 6-carboxyfluorescein-labeled RpfF promoter DNA probe (A) and RpfC promoter DNA probe (B), respectively, and incubated for 30 min at room temperature, and no binding bands were detected.

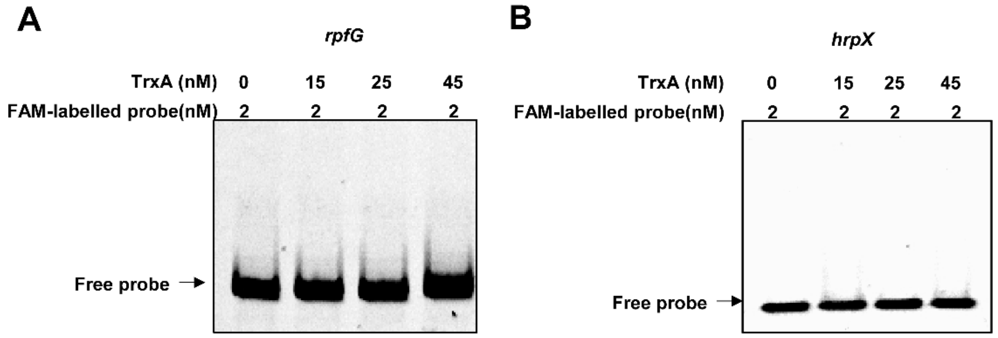

Figure. S6 TrxA-tagged proteins do not bind the promoter regions of RpfG and HrpX. TrxA-tagged proteins were subjected to EMSA with 6-carboxyfluorescein-labeled RpfG promoter DNA probe (A) and HrpX promoter DNA probe (B), respectively, and incubated for 30 min at room temperature, and no binding bands were detected.

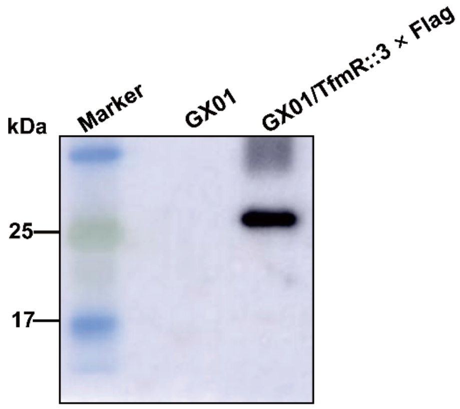

Figure. S7 Western blotting was performed on eluted TfmR::3 × Flag fusion proteins. After ChIP experiments, Western blotting was performed to detect eluted TfmR::3 × Flag fusion proteins.
