# Supplementary material for: The Transcriptional Regulator TfmR Directly Regulates Two Pathogenic Pathways in Xanthomonas oryzae pv. oryzicola
Source: Int J Mol Sci. 2024 May 28;25(11):5887. doi: 10.3390/ijms25115887 (PMC11173191; doi:10.3390/ijms25115887)
Supplement: Supplementary file 1 [file ijms-25-05887-s001.zip › Table S1.pdf]

Table S1 The ≥2-fold differentially expressed genes of the *tfmR*-mutant strain cultured in the NB medium

Function Category	Gene ID	Name	Annotation	fold change Δ*tfmR*/wt
**Amino acids biosynthesis (18)**	*XOCgx_4861*	*gltB*	glutamate synthase	-2.26
	XOCgx_1841	*trpB*	tryptophan synthase subunit beta	-2.96
	XOCgx_0577	*aroQ*	3-dehydroquinate dehydratase	2.26
	XOCgx_1843	*trpA*	tryptophan synthase subunit alpha	-2.40
	XOCgx_1571	*metB*	cystathionine gamma-synthase	-2.68
	XOCgx_1380		diaminopimelate decarboxylase	3.22
	XOCgx_2911		glutamine synthetase	-2.18
	XOCgx_2705		aspartate kinase	-2.26
	XOCgx_4862	*gltD*	glutamate synthase	-2.06
	XOCgx_3139	*metH*	5-methyltetrahydrofolate--homocysteine methyltransferase	-2.87
	XOCgx_3137	*metH*	5-methyltetrahydrofolate--homocysteine methyltransferase	-2.59
	XOCgx_0374	*metE*	5-methyltetrahydropteroyltriglutamate--homocysteine methyltransferase	-3.91
	XOCgx_2922		beta alanine—pyruvate aminotransferase	-2.14
	XOCgx_2704	*thrB*	homoserine kinase	-2.36
	XOCgx_1065	*hisC*	histidinol-phosphate aminotransferase	-2.73
	XOCgx_3138	*metH*	5-methyltetrahydrofolate--homocysteine methyltransferase	-2.19
	XOCgx_0144		aminotransferase V	-2.06
	XOCgx_1570		homoserine O-acetyltransferase	-2.22
**Nucleotides biosynthesis**	*XOCgx_3315*		type I phosphodiesterase-nucleotide pyrophosphatase family protein	-2.34

**(1)**				
**Cofactors, prosthetic groups, carriers biosynthesis (4)**	*XOCgx_4664*	*hemX*	uroporphyrin-III methyltransferase	2.10
	XOCgx_1062	*aofH*	flavin monoamine oxidase	-2.17
	XOCgx_0747		biotin synthase	2.20
	XOCgx_1375		adenosyl cobinamide kinase	-2.16
**Fatty acid and phosphatidic acid biosynthesis (10)**	*XOCgx_0101*		methylcrotonoyl-CoA carboxylase	-3.67
	XOCgx_3770	*fabG*	3-ketoacyl-ACP reductase	-2.92
	XOCgx_3771	*fabD*	malonyl CoA-ACP transacylase	-2.95
	XOCgx_4702		cardiolipin synthase	2.45
	XOCgx_0850		3-oxoacyl-ACP synthase	-2.70
	XOCgx_0851	*fabA*	3-hydroxydecanoyl-ACP dehydratase	-2.28
	XOCgx_0986	*phbB*	3-ketoacyl-ACP reductase	3.53
	XOCgx_1597		enoyl-CoA hydratase	-3.93
	XOCgx_1596		enoyl-CoA hydratase	-3.65
	XOCgx_0099		3-methylcrotonyl-CoA carboxylase	-2.05
**Degradation of polysaccharides and oligosaccharides (3)**	*XOCgx_4737*		alpha-amylase	2.05
	XOCgx_0312	*treZ*	malto-oligosyltrehalose trehalohydrolase	-2.25
	XOCgx_4732		hypothetical protein	2.59
**Degradation of small molecules (12)**	*XOCgx_1555*		peroxidase	23.61
	XOCgx_1386		4-hydroxy-2-oxovalerate aldolase	11.30
	XOCgx_1908	*hutH*	histidine ammonia-lyase	-4.30
	XOCgx_1909	*hutI*	imidazolonepropionase	-4.53
	XOCgx_1906	*hutU*	urocanate hydratase	-3.42
	XOCgx_1907	*hutG*	N-formylglutamate amidohydrolase	-4.46

	XOCgx_1910	*hutF*	N-formimino-L-glutamate deiminase	-3.79
	XOCgx_1594	*mmsA*	methylmalonate-semialdehyde dehydrogenase	-4.56
	XOCgx_0173		(2Fe-2S)-binding protein	-3.25
	XOCgx_0172		diguanylate cyclase	-2.80
	XOCgx_0163	*pobA*	4-hydroxybenzoate 3-monooxygenase	-2.08
	XOCgx_3508		Zn-dependent dipeptidase	-2.16
**Degradation of lipids (6)**	*XOCgx_1554*	*fadE*	acyl-coenzyme A dehydrogenase	18.72
	XOCgx_3135		acyl-CoA dehydrogenase	8.50
	XOCgx_0102	*ivd*	isovaleryl-CoA dehydrogenase	-3.92
	XOCgx_3036		acyl-CoA dehydrogenase	3.07
	XOCgx_1595		acyl-CoA dehydrogenase family member 8	-4.41
	XOCgx_3134		acyl-CoA dehydrogenase	6.03
**Central intermediary metabolism (5)**	*XOCgx_4593*		alkaline phosphatase D	2.89
	XOCgx_3801		hydroxyacylglutathione hydrolase	2.02
	XOCgx_2789		ribonuclease	2.57
	XOCgx_2888		succinate-semialdehyde dehydrogenase	2.29
	XOCgx_4766		sulfite exporter TauE/SafE family protein	2.37
**Energy metabolism, carbon metabolism (14)**	*XOCgx_0530*	*bfd*	bacterioferritin	12.89
	XOCgx_0459		oxidoreductase	2.50
	XOCgx_2230		amino acid oxidase	2.70
	XOCgx_1869		NADH dehydrogenase subunit M	2.11

	XOCgx_0888	*etfB*	electron transfer flavoprotein subunit beta	2.41
	XOCgx_4655		pyruvate dehydrogenase	2.24
	XOCgx_0887	*etfA*	electron transfer flavoprotein subunit alpha	2.29
	XOCgx_3421		aldehyde oxidoreductase	2.50
	XOCgx_4598		aldehyde-activating protein	2.01
	XOCgx_2014	*ccmH*	C-type cytochromebiogenesis protein	2.04
	XOCgx_2198		lactate dehydrogenase	-3540.01
	XOCgx_4522		glycosyl hydrolase	-2.07
	XOCgx_0167	*glpK*	glycerol kinase	-2.28
	XOCgx_1452		glucose kinase	-2.29
**Two component systems (10)**	*XOCgx_1445*		two-component system response regulatory protein	3.17
	XOCgx_0778		two-component system sensor-response regulator hybrid protein	2.96
	XOCgx_4036		two-component system sensor protein	2.86
	XOCgx_3026		two-component system sensor-response regulator hybrid protein	2.52
	XOCgx_4654		two-component system sensor histidine kinase	3.02
	XOCgx_4687	*ntrB*	two-component system sensor protein	-2.05
	XOCgx_4653		sigma-54-dependent Fis family transcriptional regulator	2.39
	XOCgx_1434		two-component system sensor protein	2.66
	XOCgx_3639		two-component system sensor protein	2.05

	XOCgx_3215		integrase core domain protein	-2.26
**Activators-repressors (12)**	*XOCgx_1818*		diadenosine tetraphosphate hydrolase	4.62
	XOCgx_3937		polyphosphate kinase	2.27
	XOCgx_1556		TetR family transcriptional regulator	-5249.76
	XOCgx_2185		LuxR family transcriptional regulator	-5114.05
	XOCgx_0119		MarR family transcriptional regulator	-2.07
	XOCgx_0168		hypothetical protein	-2.13
	XOCgx_0997		LysR family transcriptional regulator	-2.04
	XOCgx_2184		regulatory protein	-358.19
	XOCgx_0726		two-component system response regulator protein	2.25
	XOCgx_1911	*hutC*	histidine utilization repressor	-2.52
	XOCgx_3136		ArsR family transcriptional regulator	-3.54
	XOCgx_2210		LysR family transcriptional regulator	-2.13
**Kinases-phosphatases (5)**	*XOCgx_2195*		transcriptional regulator	-2813.41
	XOCgx_2197		transcriptional regulator	-1269.57
	XOCgx_2201		transcriptional regulator	-398.69
	XOCgx_1840		transcriptional regulator	-2.08
	XOCgx_1069		transcriptional regulator	-2.35
**Sigma factors (2)**	*XOCgx_3460*	*rrf2*	Rrf2 family transcriptional regulator	2.23
	XOCgx_3342		anti-anti-sigma factor	-2.05
	XOCgx_2088	*dnaB*	replicative DNA helicase	3.30

	XOCgx_1782		phospholipase	3.31
	XOCgx_0962	*def*	peptide deformylase	2.92
	XOCgx_0460		acyl-CoA desaturase	2.46
	XOCgx_4893		lipoprotein	2.70
	XOCgx_1422		acyl-CoA thioesterase	2.40
	XOCgx_0007		peptidase	2.30
	XOCgx_3333		peptidase C1	3.48
	XOCgx_3729		aminopeptidase N	2.28
	XOCgx_0982		amylosucrase	2.14
**Macromolecule metabolism (26)**	*XOCgx_2054*		peptidase S10	2.24
	XOCgx_4859		DNA topoisomerase	2.27
	XOCgx_3132		acetylhydrolase	2.09
	XOCgx_0740		glycosyl transferase, group 2 family protein	3.13
	XOCp0037	*yecR*	YecR-like lipoprotein	-2.77
	XOCgx_2199		DEAD/DEAH box helicase	-16470.20
	XOCgx_2200		helicase, putative	-8378.36
	XOCgx_4943	*glpQ*	glycerophosphodiester phosphodiesterase	-2.17
	XOCgx_2187	*repA*	replication protein A	-1937.86
	XOCgx_4369	*tdcF*	N-acyl-L-amino acid amidohydrolase	-2.02
	XOCgx_1064	*tdcF*	translation initiation inhibitor, yjgF family	-2.84
	XOCgx_2188	*repC*	replication protein C	-509.90

	XOCgx_1285		lipoprotein	-2.11
	XOCgx_0310	*glgA*	glycogen synthase	-2.00
	XOCgx_3445	*tag*	DNA-3-methyladenine glycosylase I	-2.48
	XOCgx_2822		ATP-dependent DNA ligase	-2.36
**Cell structure (38)**	*XOCgx_0804*		membrane protein	5.62
	XOCgx_4204		membrane protein	9.51
	XOCgx_4208		membrane protein	6.20
	XOCgx_1788		peptidoglycan-associated outer membrane lipoprotein	4.06
	XOCgx_0989		porin	12.10
	XOCgx_3437		membrane protein	3.62
	XOCgx_3855		membrane protein	4.53
	XOCgx_1793		membrane protein	2.78
	XOCgx_1923		cell envelope biogenesis protein TonB	4.16
	XOCgx_0133		membrane protein	3.15
	XOCgx_2810		N-acetylmuramoyl-L-alanine amidase	2.64
	XOCgx_1120		membrane protein	2.87
	XOCgx_0038		outer membrane protein	2.28
	XOCgx_3251		membrane protein	2.35
	XOCgx_1121		membrane protein	2.27
	XOCgx_1720	*rseP*	zinc metalloprotease	2.23
	XOCgx_3873		membrane protein	2.51

	XOCgx_4234	*mrdB*	rod shape-determining protein RodA	2.34
	XOCgx_4659		membrane protein	2.26
	XOCgx_0065		membrane protein	2.10
	XOCgx_3117	*oprO_P*	phosphate-selective porin OprO and OprP	2.12
	XOCgx_0751		fasciclin	2.76
	XOCgx_3475	*murU*	mannose-1-phosphate guanylyltransferase	2.21
	XOCgx_3229		membrane protein	2.04
	XOCgx_3481		membrane protein	2.00
	XOCgx_3473		membrane protein	2.76
	XOCgx_0006		membrane protein	2.04
	XOCgx_1642		membrane protein	2.23
	XOCgx_3309		membrane protein	2.22
	XOCgx_0803		membrane protein	2.88
	XOCgx_0651		murein transglycosylase	-2.39
	XOCgx_1889		Oar protein	-2.38
	XOCgx_3443		membrane protein	-2.37
	XOCgx_2989		TonB-dependent outer membrane receptor	-3.88
	XOCgx_2636		membrane protein	-2.51
	XOCgx_1842		membrane protein	-2.05
	XOCgx_0967		membrane protein	-2.80
	XOCgx_3444		membrane protein	-2.38
	XOCgx_1747		ligand-gated channel	6.78

**Cellular processes and community (53)**				
	XOCgx_1385		TonB-dependent receptor	5.55
	XOCgx_0390	*cysJ*	iron-uptake factor	5.80
	XOCgx_2651	*feoB*	iron transporter FeoB	3.59
	XOCgx_4048		hemin transporter	6.47
	XOCgx_1383		vibrioferrin biosynthesis protein PvsB	3.88
	XOCgx_0922		membrane protein	3.26
	XOCgx_3120	*pstC*	phosphate ABC transporter, permease protein PstC	2.99
	XOCgx_0921		membrane protein	2.71
	XOCgx_3121	*pstA*	phosphate ABC transporter, permease protein PstA	2.72
	XOCgx_1381		iron transporter	3.79
	XOCgx_2725		sodium transporter	2.75
	XOCgx_1034		biopolymer transporter Tol	2.42
	XOCgx_3876		TonB-dependent receptor	2.78
	XOCgx_4512	*mgtE*	magnesium transporter	3.08
	XOCgx_2652	*feoA*	iron transporter FeoA	4.32
	XOCgx_3802		lytic transglycosylase	2.43
	XOCgx_0300		ion channel protein	2.47
	XOCgx_2286	*lolC*	lipoprotein releasing system transmembrane protein	2.42
	XOCgx_0984		sugar transporter	2.61
	XOCgx_4099	*ftsW*	cell division protein FtsW	2.17

	XOCgx_2052		inorganic phosphate transporter	2.17
	XOCgx_2945		PTS fructose transporter subunit IIBC	2.10
	XOCgx_1384		vibrioferrin biosynthesis protein PvsA	3.61
	XOCgx_0987		citrate transporter	3.17
	XOCgx_2838		MFS transporter	2.26
	XOCgx_1420	*kup*	potassium uptake protein	2.19
	XOCgx_4715		amino acid ABC transporter permease	2.45
	XOCgx_2973		TonB-dependent receptor	2.20
	XOCgx_0305		ABC transporter substrate-binding protein	2.52
	XOCgx_3745		TonB-dependent receptor	2.39
	XOCgx_1426	*tolA*	cell envelope integrity/translocation protein TolA	2.01
	XOCgx_4095	*ftsQ*	cell division protein FtsQ	2.04
	XOCgx_1382		transport protein	2.76
	XOCgx_1002		C4-dicarboxylate transporter	2.28
	XOCgx_1656		cation transporter	2.51
	XOCgx_3862		cell wall hydrolase	2.66
	XOCgx_0835		ABC transporter permease	2.81
	XOCgx_0988		Mg++-citrate complex transporter	5.98
	XOCgx_1749	*bfr*	bacterioferritin	-9.45
	XOCgx_0531	*bfr*	bacterioferritin	-8.15

	XOCgx_1557		TonB-dependent receptor plug domain protein	-2.38
	XOCgx_0999	*citA*	citrate-proton symporter	-3.31
	XOCgx_2988		TonB-dependent receptor	-3.02
	XOCgx_1036		TonB-dependent receptor	-2.26
	XOCgx_1633		cell shape determination protein CcmA	-2.48
	XOCgx_1530		TonB-dependent receptor	-2.06
	XOCgx_4339		TonB-dependent outer membrane receptor	-2.19
	XOCgx_2996		TonB-dependent receptor	-2.14
	XOCgx_0998		molybdenum ABC transporter substrate-binding protein	-3.71
	XOCgx_3258		energy transducer TonB	-2.35
	XOCgx_2189		peptide transporter	-147.24
	XOCgx_2849	*parB*	chromosome partitioning protein	-2.34
**Pathogenicity, virulence, invasion and adaptation (72)**	*XOCgx_3854*		NADPH-dependent 2,4-dienoyl-CoA reductase	25.37
	XOCgx_4049		hemin transporter HemP	15.42
	XOCgx_0419		entericidin	4.20
	XOCgx_4274		glucosyltransferase	2.60
	XOCgx_1270		competence protein	2.49
	XOCgx_4410		virulence factor BrkB	4.13
	XOCgx_2610	*tsr*	methyl-accepting chemotaxis protein	2.94
	XOCgx_1427	*tolB*	translocation protein TolB	2.44

	XOCgx_2609	*tsr*	methyl-accepting chemotaxis protein	2.94
	XOCgx_4543		fatty acyl CoA synthetase	2.37
	XOCgx_0420		entericidin	3.54
	XOCgx_4804		attachment protein	4.92
	XOCgx_3051	*gumE*	exopolysaccharide xanthan biosynthesis polymerase GumE	2.32
	XOCgx_4656		Ax21 family protein	2.04
	XOCgx_4541		membrane protein involved in xanthomonadin export	2.32
	XOCgx_2726		1,4-beta-D-glucan glucohydrolase	2.18
	XOCgx_0294	*hpa1*	type III secretion harpin protein Hpa1	4.36
	XOCgx_1216		sulfur deprivation response regulator	2.17
	XOCgx_2050		hemolysin	2.23
	XOCgx_4791		CsbD family protein	2.72
	XOCgx_0983		TonB-dependent receptor	2.17
	XOCgx_2959	*secD*	preprotein translocase subunit SecD	2.07
	XOCgx_2508	*rpoN*	RNA polymerase sigma-54 factor	2.29
	XOCgx_4235		penicillin-binding protein 2	2.09
	XOCgx_1748		penicillin acylase	2.11
	XOCgx_4876		ABC transporter permease	2.07
	XOCgx_1856	*secG*	preprotein translocase subunit SecG	2.02
	XOCgx_0708		chemotaxis protein, putative	2.21
	XOCgx_0942	*xpsN*	general secretion pathway protein GspN	2.11

	XOCgx_4713		ergothioneine biosynthesis protein EgtB	2.32
	XOCgx_1567		hemolysin III	2.43
	XOCgx_0272	*xopF1*	type III effector protein XopF1	2.03
	XOCgx_2049	*exoD*	exopolysaccharide synthesis protein exoD	2.45
	XOCgx_2126		ABC transporter ATP-binding protein	2.17
	XOCgx_0953	*xagB*	glycosyltransferase	2.26
	XOCgx_0238	*tatC*	preprotein translocase subunit TatC	2.28
	XOCgx_3050	*gumF*	exopolysaccharide xanthan biosynthesis acetyltransferase GumF	2.05
	XOCgx_0274	*hrpE*	type III secretion pilin HrpE	2.30
	XOCgx_2048		general stress protein	2.59
	XOCgx_4597		transglycosylase	2.66
	XOCgx_3821	*phoR*	two-component system sensor protein PhoR	2.23
	XOCgx_2527		flagellar hook-basal body complex protein (FliE)	2.49
	XOCgx_2399		serine protease	2.34
	XOCgx_0421		stress response protein, putative	6.29
	XOCgx_0981		dimethylallyltransferase	2.09
	XOCgx_0952	*xagA*	hypothetical protein	2.06
	XOCgx_2257		hemolysin D	2.14
	XOCgx_4471	*xopW*	type III effector protein XopW	2.02
	XOCgx_4247		hemolysin secretion protein D	2.27

	XOCgx_0271	*hpa3*	Hpa3 protein	2.59
	XOCgx_2032		lipopolysaccharide biosynthesis protein	2.19
	XOCgx_4805	*trxA*	thioredoxin	6.93
	XOCgx_0284	*hrpB1*	type III secretion protein HrpB1	2.59
	XOCgx_2561		Rhs element Vgr protein	3.25
	XOCp0024	*virB9*	Type IV secretion system protein VirB9	-2.93
	XOCp0021	*virB1*	Type IV secretion system protein VirB1	-2.73
	XOCp0023	*virB10*	Type IV secretion system proteinj VirB10	-2.78
	XOCgx_0620		glutathione S-transferase	-2.53
	XOCp0022	*virB11*	Type IV secretion system protein VirB11	-2.47
	XOCgx_4691		undecaprenyl-diphosphatase	-2.56
	XOCgx_0022		cellulase	-2.92
	XOCgx_2486	*flgM*	negative regulator of flagellin synthesis FlgM	-2.20
	XOCp0029	*virB5*	type IV secretion system protein VirB5	-2.46
	XOCp0025	*virB8*	Type IV secretion system protein VirB8	-2.22
	XOCgx_2614	*mcp*	methyl-accepting chemotaxis protein	-2.35
	XOCgx_3343		two-component system sensor protein	-2.05
	XOCp0028	*virB7*	type IV secretion system protein VirB7-like protein	-2.14
	XOCgx_4574		universal stress protein UspA	-2.40
	XOCgx_1068	*azu*	azurin	-2.70
	XOCgx_2406	*yapH*	filamentous hemagglutinin-related protein	-2.19

	XOCgx_3522		beta-lactamase	-2.29
	XOCgx_0375		protein-S-isoprenylcysteine methyltransferase	-3.49
**Mobile genetic elements (Mobilomes) (47)**	*XOCgx_0454*		transposase, partial	3.01
	XOCgx_1041		transposase, putative	2.44
	XOCgx_1588		integrase, partial	2.49
	XOCgx_4749		transposase	2.24
	XOCgx_1042		transposase	2.02
	XOCgx_2695		transposase	89.13
	XOCgx_2985		transposase	3.85
	XOCgx_0657		transposase, partial	2.00
	XOCgx_4788		transposase	-2.90
	XOCgx_2183		integrase	-5337.48
	XOCp0049		membrane protein	-2.16
	XOCgx_4787		transposase	-2.55
	XOCgx_2193	*trbL*	conjugal transfer protein TrbL	-1807.57
	XOCgx_1345		transposase	-2.50
	XOCgx_4864		transposase	-2.12
	XOCgx_2435		transposase	-2.29
	XOCgx_0219		transposase	-2.24
	XOCp0038	*tnpA*	transposase	-2.30
	XOCgx_2373		transposase	-3.39

	XOCgx_4500		transposase	-2.91
	XOCgx_2190	*traJ*	conjugal transfer protein TraJ	-359.06
	XOCgx_2305		transposase	-1224.82
	XOCgx_0655		DDE endonuclease	-2.40
	XOCgx_2369		transposase	-2.14
	XOCgx_1499		transposase	-2.12
	XOCgx_2191	*trbJ*	conjugal transfer protein TrbJ	-158.20
	XOCgx_0471		transposase	-2.27
	XOCgx_0842		transposase	-2.56
	XOCgx_4681		transposase	-2.37
	XOCgx_2371		IS1113 transposase	-2.03
	XOCgx_4301		transposase	-2.03
	XOCgx_0045		transposase	-2.12
	XOCgx_4903		transposase	-2.62
	XOCgx_2186		plasmid-related protein	-82.50
	XOCgx_3217		transposase	-2.24
	XOCgx_1433		transposase	-2.04
	XOCgx_2390		transposase	-2.26
	XOCgx_0220		transposase	-2.18
	XOCgx_4340		transposase	-2.07
	XOCgx_2192	*trbK*	entry/exclusion protein TrbK	-48.99
	XOCgx_2000		ISXoo3 transposase orfB	-2.14

	XOCgx_1954		transposase	-4.21
	XOCgx_1659		ISXo1 transposase, IS5 family	-4.21
	XOCgx_2263		transposase	-4.21
	XOCgx_1943		transposase	-4.21
	XOCgx_1978		conjugal transfer protein TraD	-2.38
	XOCgx_0341		transposase	-11.69
**Poorly characterized or undefined category (108)**	*XOCgx_0813*		hypothetical protein	5.09
	XOCgx_1691		DUF2147 domain-containing protein	4.90
	XOCgx_0776		hypothetical protein	7.58
	XOCgx_4080		protease	6.25
	XOCgx_2324		DUF3300 domain-containing protein	3.75
	XOCgx_4207		hypothetical protein	5.65
	XOCgx_2142		hypothetical protein	3.11
	XOCgx_4385		EF hand domain-containing protein	3.60
	XOCgx_4925		DUF2384 domain-containing protein	3.17
	XOCgx_3484		DUF3108 domain-containing protein	2.67
	XOCgx_3483		DUF3108 domain-containing protein	3.01
	XOCgx_0614		hypothetical protein	4.80
	XOCgx_4073		hypothetical protein	3.46
	XOCgx_4079		glyoxalase	6.34
	XOCgx_0777		membrane protein, putative	6.35

	XOCgx_1635		hypothetical protein	3.13
	XOCgx_3033		hypothetical protein	2.72
	XOCgx_4143		isoaspartyl peptidase/L-asparaginase	4.16
	XOCgx_1411		DUF3011 domain-containing protein	2.64
	XOCgx_2104		hypothetical protein	2.34
	XOCgx_4668		hypothetical protein	5.12
	XOCgx_4209		DUF2242 domain-containing protein	2.78
	XOCgx_3573		hypothetical protein	2.60
	XOCgx_1745		right-handed parallel beta-helix repeat-containing protein	2.06
	XOCgx_4124		hypothetical protein	4.78
	XOCgx_1118		DUF4381 domain-containing protein	3.09
	XOCgx_4072		sulfotransferase	2.31
	XOCgx_2949		FAD-dependent oxidoreductase	2.32
	XOCgx_1952		hypothetical protein	2.67
	XOCgx_4158		hypothetical protein	2.48
	XOCgx_3035		LEA type 2 family protein	2.57
	XOCgx_4652		hypothetical protein	2.32
	XOCgx_2858		hypothetical protein	2.40
	XOCgx_3011		DUF885 family protein	2.09
	XOCgx_4033	*lemA*	LemA family protein	2.21
	XOCgx_4764		membrane protein, putative	3.42

	XOCgx_0404		hypothetical protein	2.21
	XOCgx_0094		hypothetical protein	2.39
	XOCgx_4032		dehydrogenase	2.58
	XOCgx_4809		peptidase	2.26
	XOCgx_3738		hypothetical protein	2.08
	XOCgx_1465		tetratricopeptide repeat domain protein	2.02
	XOCgx_3279		hypothetical protein	2.33
	XOCgx_1732		spore coat protein U	2.04
	XOCgx_0584		DUF3106 domain-containing protein	2.15
	XOCgx_3474		aminoglycoside phosphotransferase	2.01
	XOCgx_4926		enterochelin esterase	3.61
	XOCgx_0812		hypothetical protein	2.51
	XOCgx_3032		hypothetical protein	3.08
	XOCgx_4222		hypothetical protein	2.97
	XOCgx_4627		hypothetical protein	2.05
	XOCgx_2648		secreted protein, putative	2.71
	XOCgx_2288		hypothetical protein	3.16
	XOCgx_0066		hypothetical protein	3.74
	XOCgx_4144		hypothetical protein	6.16
	XOCgx_4396		hypothetical protein	3.95
	XOCgx_3487		hypothetical protein	2.14
	XOCgx_2162		hypothetical protein	2.02

	XOCgx_2650		membrane protein, putative	2.03
	XOCgx_0725		hypothetical protein	2.11
	XOCgx_0500		hypothetical protein	2.26
	XOCgx_0234		hypothetical protein	2.13
	XOCgx_1359		hypothetical protein	4.09
	XOCgx_3021		hypothetical protein	3.22
	XOCgx_4470		hypothetical protein	23.29
	XOCgx_0749		hypothetical protein	5.83
	XOCgx_0443		hypothetical protein	2.00
	XOCgx_0137		membrane protein, putative	2.19
	XOCgx_4790		hypothetical protein	8.22
	XOCp0020		hypothetical protein	-2.37
	XOCgx_3374		hypothetical protein	-3.07
	XOCgx_2987		Secreted protein	-2.57
	XOCgx_1503	*aga*	N(4)-(Beta-N-acetylglucosaminyl)-L-asparaginase	-2.49
	XOCgx_2202		AAA family ATPase	-4686.40
	XOCgx_2613		hypothetical protein	-2.47
	XOCgx_4899		immunity 52 family protein	-2.02
	XOCgx_4162		nodulin 21-related protein	-2.21
	XOCgx_2912		glutamine amidotransferase	-2.43
	XOCgx_4896		DUF4123 domain-containing protein	-2.50

	XOCgx_2770		quercetin 2,3-dioxygenase	-2.74
	XOCgx_1052	*atuG*	short-chain dehydrogenase	-2.18
	XOCgx_3278		DUF2867 domain-containing protein	-2.28
	XOCgx_2194		type I toxin-antitoxin system ptaRNA1 family toxin	-247.50
	XOCgx_2368		hypothetical protein	-2.24
	XOCgx_0372		NADH-dependent FMN reductase	-4.56
	XOCgx_0058		DUF4272 domain-containing protein	-2.30
	XOCgx_1500		hypothetical protein	-2.26
	XOCgx_1199		hypothetical protein	-2.38
	XOCgx_0712		hypothetical protein	-2.97
	XOCgx_0409		zinc-dependent peptidase	-2.01
	XOCgx_4569		3-hydroxyisobutyrate dehydrogenase	-2.58
	XOCgx_4814		hypothetical protein	-2.12
	XOCgx_2693		tannase	-2.14
	XOCgx_0658		hypothetical protein	-8.39
	XOCgx_2774		hypothetical protein	-2.15
	XOCgx_4837		hypothetical protein	-2.11
	XOCgx_0373		DUF1852 domain-containing protein	-3.72
	XOCgx_2196		hypothetical protein	-55.76
	XOCgx_3975		hypothetical protein	-3.15
	XOCgx_4263		hypothetical protein	-2.87

	XOCgx_4363		hypothetical protein	-2.31
	XOCgx_1453		glucokinase, partial	-2.02
	XOCp0041		hypothetical protein	-2.08
	XOCgx_1977		hypothetical protein	-2.46
	XOCgx_2877		hypothetical protein	-3.02
	XOCgx_1989		hypothetical protein	-2.00
	XOCgx_0625		cupin	-2.04
	XOCgx_0221		hypothetical protein	-2.01
