# Supplementary material for: The Transcriptional Regulator TfmR Directly Regulates Two Pathogenic Pathways in Xanthomonas oryzae pv. oryzicola
Source: Int J Mol Sci. 2024 May 28;25(11):5887. doi: 10.3390/ijms25115887 (PMC11173191; doi:10.3390/ijms25115887)
Supplement: Supplementary file 1 [file ijms-25-05887-s001.zip › Table S2.pdf]

Table S2 Bacterial strains and plasmids used in this work

**Strains or plasmids**	**Relevant characteristics**	**Reference or source**
E. coli strains		
DH5α	Φ80△*lacZM15 recA1 endA1 deoR*	Gibco BRL, Life Technologies
BL21(DE3)	F^- *ompT gal dcm lon* $hsdS_B$ (r^-_B m^-_B) λ(DE3)	Novagen
BL21/pET-32a-TfmR	BL21(DE3) harboring recombinant plasmid pET-32a-TfmR	This work
Xanthomonas oryzae pv. *oryzicola* trains		
GX01	Wild-type strain, isolated from Guangxi, China, Rif^r	Author's lab collection
Δ*tfmR*	As GX01, but XOCgx_1556 (*tfmR*) gene deleted, non-polar effect. Rif^r	This work
CΔ*tfmR*	Δ*tfmR* harboring the recombinant plasmid pKC*tfmR* Rif^r Kan^r	This work
GX01/TfmR::3×Flag	As GX01, but chromosomally encoding a 3×Flag fused TfmR protein. Rif^r	This work
Δ*tfmR*/pKG	Δ*tfmR* harboring the recombinant plasmid pKG. Rif^r Kan^r	This work
Δ*tfmR* /pXUK	Δ*tfmR* harboring the plasmid pXUK. Rif^r Kan^r	This work
Δ*tfmR* /pKX	Δ*tfmR* harboring the recombinant plasmid pKX. Rif^r Kan^r	This work
GX01/pGUS*rpfG*	GX01 harboring the reporter plasmid pGUS*rpfG*. Rif^r Tet^r	This work
GX01/pGUS*hrpG*	GX01 harboring the reporter plasmid pGUS*hrpG*. Rif^r Tet^r	This work
GX01/pGUS*hrpX*	GX01 harboring the reporter plasmid pGUS*hrpX*. Rif^r Tet^r	This work
Δ*tfmR*/pGUS*rpfG*	Δ*tfmR* harboring the reporter plasmid pGUS*rpfG*. Rif^r Tet^r	This work
Δ*tfmR*/pGUS*hrpG*	Δ*tfmR* harboring the reporter plasmid pGUS*hrpG*. Rif^r Tet^r	This work
Δ*tfmR*/pGUS*hrpX*	Δ*tfmR* harboring the reporter plasmid pGUS*hrpX*. Rif^r Tet^r	This work
ΔXOCgx_0458	As GX01, but XOCgx_0458 gene deleted, non-polar effect. Rif^r	This work
ΔXOCgx_1016	As GX01, but XOCgx_1016 gene deleted, non-polar effect. Rif^r	This work
ΔXOCgx_2380	As GX01, but XOCgx_2380 gene deleted, non-polar	This work

	effect. Rif^r	
ΔXOCgx_2451	As GX01, but XOCgx_2451 gene deleted, non-polar effect. Rif^r	This work
ΔXOCgx_2936	As GX01, but XOCgx_2936 gene deleted, non-polar effect. Rif^r	This work
ΔXOCgx_3699	As GX01, but XOCgx_3699 gene deleted, non-polar effect. Rif^r	This work
ΔXOCgx_3726	As GX01, but XOCgx_3726 gene deleted, non-polar effect. Rif^r	This work
ΔXOCgx_4067	As GX01, but XOCgx_4067 gene deleted, non-polar effect. Rif^r	This work
Plasmids		
pXUK	Adapted from an endogenous plasmid in Xoc strain GX01. Kan^r	[26]
pKC*tfmR*	pXUK cloned into a 606-bp DNA fragment containing the full-length *tfmR* gene of *Xoc* strain. Kan^r	This work
pKG	pXUK cloned into a 1137-bp DNA fragment containing the full-length *rpfG* gene of *Xoc* strain. Kan^r	This work
pKX	pXUK cloned into a 1431-bp DNA fragment containing the full-length *hrpX* gene of *Xoc* strain. Kan^r	This work
pRK2073	Helper plasmid, Tra$^+$, Mob$^+$, ColE1, Spc^r	[45]
pET-32a	Expression vector, allow the production of fusion proteins containing amino terminal thioredoxin-tagged and carboxyl-terminal 6×His-tagged sequences. Amp^r	Novagen
pET-32a-TfmR	pET-32a cloned into a 606-bp DNA fragment of the *tfmR* gene coding sequence.	This work
pK18*mobsacB*	pUC18 derivative, *lacZa*, *sacB*, Kan^r, *mob* site. Allelic exchange vector (Suicidal vector carrying *sacB* gene for mutagenesis)	[44]
pKΔ*tfmR*	pK18mobsacB containing upstream and downstream fragments of the *tfmR* coding region. Kan^r	This work
pGUS*rpfG*	pLAFR6 containing a *rpfG* promoter-*gusA* fusion fragment. Tet^r	This work
pGUS*hrpG*	pLAFR6 containing an *hrpG* promoter-*gusA* fusion fragment. Tet^r	This work
pGUS*hrpX*	pLAFR6 containing an *hrpX* promoter-*gusA* fusion fragment. Tet^r	This work
pKTfmR::Flag	pK18*mobsacB* containing fragment composing 606-bp TfmR-coding sequence, 66-bp 3×Flag-coding sequence sequence, 3-bp stop codon, 425-bp upstream of the *Xoc tfmR* and 309-bp downstream of the *Xoc tfmR*.	This work
pKΔXOCgx_0458	pK18mobsacB containing upstream and downstream fragments of the XOCgx_0458 coding region. Kan^r	This work
pKΔXOCgx_1016	pK18mobsacB containing upstream and downstream	This work

	fragments of the XOCgx_1016 coding region. Kan^r	
pKΔXOCgx_2380	pK18mobsacB containing upstream and downstream fragments of the XOCgx_2380 coding region. Kan^r	This work
pKΔXOCgx_2451	pK18mobsacB containing upstream and downstream fragments of the XOCgx_2451 coding region. Kan^r	This work
pKΔXOCgx_2936	pK18mobsacB containing upstream and downstream fragments of the XOCgx_2936 coding region. Kan^r	This work
pKΔXOCgx_3699	pK18mobsacB containing upstream and downstream fragments of the XOCgx_3699 coding region. Kan^r	This work
pKΔXOCgx_3726	pK18mobsacB containing upstream and downstream fragments of the XOCgx_3699 coding region. Kan^r	This work
pKΔXOCgx_4067	pK18mobsacB containing upstream and downstream fragments of the XOCgx_4067 coding region. Kan^r	This work

Rif^r, Kan^r, Tet^r, Amp^r, and Spc^r indicate resistance to rifampicin, kanamycin, tetracycline, ampicillin, and spectinomycin, respectively.

References

26. Zhu, P.-C.; Li, Y.-M.; Yang, X.; Zou, H.-F.; Zhu, X.-L.; Niu, X.-N.; Xu, L.-H.; Jiang, W.; Huang, S.; Tang, J.-L.; et al. Type VI secretion system is not required for virulence on rice but for inter-bacterial competition in *Xanthomonas oryzae* pv. *oryzicola*. *Research in Microbiology* **2020**, 171, 64-73, doi:https://doi.org/10.1016/j.resmic.2019.10.004.
44. Schäfer, A.; Tauch, A.; Jäger, W.; Kalinowski, J.; Thierbach, G.; Pühler, A. Small mobilizable multi-purpose cloning vectors derived from the *Escherichia coli* plasmids pK18 and pK19: selection of defined deletions in the chromosome of *Corynebacterium glutamicum*. *Gene* **1994**, 145, 69-73, doi:https://doi.org/10.1016/0378-1119(94)90324-7.
45. Leong, S.A.; Ditta, G.S.; Helinski, D.R. Heme biosynthesis in Rhizobium. Identification of a cloned gene coding for delta-aminolevulinic acid synthetase from *Rhizobium meliloti*. J*ournal of Biological Chemistry* **1982**, 257, 8724-8730, doi:10.1016/s0021-9258(18)34188-7.
