# Supplementary material for: The Transcriptional Regulator TfmR Directly Regulates Two Pathogenic Pathways in Xanthomonas oryzae pv. oryzicola
Source: Int J Mol Sci. 2024 May 28;25(11):5887. doi: 10.3390/ijms25115887 (PMC11173191; doi:10.3390/ijms25115887)
Supplement: Supplementary file 1 [file ijms-25-05887-s001.zip › Table S3.pdf]

Table S3 Primers used in this study

**Primers**	**Nucleotide sequence (5′ →3′)**	**The amplified fragment or the utilization**
L*tfmR*-F<br>L*tfmR*-R	CGGGATCC CCAGCACGATCAGATCATCGGT<br>GCTCTAGA GGCGCGATTTAACCATACGC	425-bp DNA sequence upstream of *tfmR* (XOCgx_1556), used for construction of *tfmR* deletion mutant.
R*tfmR*-F<br>R*tfmR*-R	GCTCTAGA GCTGACGTGGTGAGATCGAA<br>CCAAGCTT AGCTACTTTGATTTTGGCGTG	309-bp DNA sequence downstream of *tfmR* (XOCgx_1556), used for construction of *tfmR* deletion mutant.
C*tfmR*-F<br>C*tfmR*-R	CCAAGCTT ATGAACGACACCACCGACTCCA<br>GCTCTAGA TCACCCAGGAATCAACGTCTGGATCA	606-bp DNA fragment containing the full length *tfmR* gene. Used for complemented tests.
pET32*tfmR*-F<br>pET32*tfmR*-R	CGGGATCC ATGAACGACACCACCGACTCCA<br>CCAAGCTT CCCAGGAATCAACGTCTGGATCA	606-bp DNA fragment of the *tfmR*-coding sequence. Cloned into expression vector pET-32a for protein overproduction.
L*tfmR*-flag-F<br>L*tfmR*-flag-R	CGGGATCC CCAGCACGATCAGATCATCGGT<br>TCATGATCTTTATAATCACCGTCATGGTCTTTGTAGTCCCCAGGAATCAACGTCTGGAT	1069-bp DNA fragment containing 38-bp Flag-coding sequence, 606-bp DNA fragmen of *tfmR* ORF sequence and 425-bp DNA sequence upstream of *of the tfmR* start codon. Used for constructing *Xoc* strain producing TfmR::3×Flag protein.
R*tfmR*-flag-F<br>R*tfmR*-flag-R	ACGGTGATTATAAAGATCATGACATCGACTACAAGGATGACGATGACAAGTGAGCTGACGTGGTGAGATCG<br>CCAAGCTT AGCTACTTTGATTTTGGCGTG	365-bp DNA fragment containing 50-bp Flag-coding sequence, 3-bp stop codon and 309-bp downstream sequence of the *tfmR* stop codon. Used for constructing *Xoc* strain producing TfmR::3×Flag protein.
RpfG-EM-F<br>RpfG-EM-R	TGCTGCAGCTGAAGGTCAGG<br>FAM-CCTCCTGAAACATCCTGCAT	193-bp DNA fragment composed of 173-bp upstream and 20-bp downstream sequences of *rpfG* start codon. Used for EMSA assays.
RpfF-EM-F<br>RpfF-EM-R	TTGGCAAGATCCTGCGCCG<br>FAM-GGGTTGAACAGCAGACATGG	241-bp DNA fragment composed of 223-bp upstream and 18-bp downstream sequences of *rpfF* start codon. Used for EMSA assays.
RpfC-EM-F<br>RpfC-EM-R	GTTTCGGCAGTGAGGCCATG<br>FAM-CCATGTCAGTGGAGACTTCAT	276-bp DNA fragment composed of 255-bp upstream and 21-bp downstream sequences of *rpfC* start codon. Used for EMSA assays.
RpfG-NOEM-F<br>RpfG-NOEM-R	TGCTGCAGCTGAAGGTCAGG<br>CCTCCTGAAACATCCTGCAT	193-bp DNA fragment composed of 173-bp upstream and 20-bp downstream sequences of *rpfG* start codon. Used for 次 competition experiments for unlabeled probes in EMSA assays.
HrpX-EM-F<br>HrpX-EM-R	TGTTCTGTGCACGACACTGCTG<br>FAM-CGCAGAGATCGCTGCAAAGT	246-bp DNA fragment composed of 210-bp upstream and 36-bp downstream sequences of *hrpX* start codon. Used for EMSA assays.
HrpX-NOEM-F<br>HrpX-NOEM-R	TGTTCTGTGCACGACACTGCTG<br>CGCAGAGATCGCTGCAAAGT	246-bp DNA fragment composed of 210-bp upstream and 36-bp downstream sequences of *hrpX* start codon. Used for competition experiments for unlabeled probes in EMSA assays.

HutG-EM-F<br>HutG-EM-R	GTTGGCAGCTCTCATGCAGC<br>FAM-CGTTCATTGCTGCGCTCCAT	191-bp DNA fragment composed of 184-bp upstream and 7-bp downstream sequences of *hutG* start codon. Used for EMSA assays.
RpfG-ivtF<br>RpfG-ivtR	TGCTGCAGCTGAAGGTCAGG<br>GATGTCCTCGATGACATGGCG	323-bp DNA fragment containing 173-bp upstream of *rpfG* start codon and 150-bp *rpfG*-coding sequence. Used for In vitro transcription assays.
fRpfG-ivtF<br>fRpfG-ivtR	ATGCAGGATGTTTCAGGAGGTC<br>GATGTCCTCGATGACATGGCG	150-bp *rpfG*-coding sequence. Used for In vitro transcription assays.
HrpX-ivtF<br>HrpX-ivtR	TGTTCTGTGCACGACACTGCTG<br>TCATCGTCGGCTCCATCGGC	371-bp DNA fragment containing 210-bp upstream of *hrpX* start codon and 161-bp *hrpX*-coding sequence. Used for In vitro transcription assays.
fHrpX-ivtF<br>fHrpX-ivtR	ATGATCCTTTCGACCTACTTTGCA<br>TCATCGTCGGCTCCATCGGC	161-bp *hrpX*-coding sequence. Used for In vitro transcription assays.
HutG-ivtF<br>HutG-ivtR	GTTGGCAGCTCTCATGCAGC<br>CACCCACCAATCGGTATCGC	334-bp DNA fragment containing 184-bp upstream of *hutG* start codon and 150-bp *hutG*-coding sequence. Used for In vitro transcription assays.
RpfG-chipF<br>RpfG-chipR	TGCTGCAGCTGAAGGTCAGG<br>CCTCCTGAAACATCCTGCAT	193-bp DNA fragment containing 173-bp upstream sequence of *rpfG* start codon and 20-bp *rpfG*-coding sequence, used for ChIP-qPCR assays.
HrpX-chipF<br>HrpX-chipR	TGTTCTGTGCACGACACTGCTG<br>CGCAGAGATCGCTGCAAAGT	246-bp DNA fragment containing 210-bp upstream sequence of *hrpX* start codon and 36-bp *hrpX*-coding sequence, used for ChIP-qPCR assays.
HutG-chipF<br>HutG-chipR	GTTGGCAGCTCTCATGCAGC<br>CGTTCATTGCTGCGCTCCAT	191-bp DNA fragment containing 184-bp upstream sequence of *hutG* start codon and 7-bp *hutG*-coding sequence, used for ChIP-qPCR assays.
RpfG-PGUSF<br>RpfG-PGUSR	CGGAATTC TGCTGCAGCTGAAGGTCAGG<br>CGGGATCC CATAGCGGTTGCTCCTCTATTC	176-bp DNA fragment containing 173-bp upstream sequence of *rpfG* start codon and 3-bp start codon sequence, used for GUS enzyme activity tests.
HrpX-PGUSF<br>HrpX-PGUSR	CGGAATTC TGTTCTGTGCACGACACTGCTG<br>CGGGATCC CATGCCGGTCTCTCTCTTGG	213-bp DNA fragment containing 210-bp upstream sequence of *hrpX* start codon and 3-bp start codon sequence, used for GUS enzyme activity tests.
HrpG-PGUSF<br>HrpG-PGUSR	CGGAATTC GATGAGCGCGAGTTAAAGCCAA<br>CGGGATCC CATCAGGTGGGCGTCCCG	239-bp DNA fragment containing 236-bp upstream sequence of *hrpG* start codon and 3-bp start codon sequence, used for GUS enzyme activity tests.
16SF<br>16SR	GCCTAACACATGCAAGTCGAACGGC<br>AATATTCCCCACTGCTGCCTCCCG	325-bp DNA fragment of the 16S rDNA sequence, used for RT-qPCR.
rpfG-F<br>*rpfG*-R	ATGACCAGATGTCTGCGCGGAC<br>GAATTCCAGACCGTCCATGCCG	173-bp DNA fragment of the *rpfG*-coding sequence, used for RT-qPCR.
rpfC-F<br>*rpfC*-R	GAAACAACGTCTGTCAGGCCGG<br>GATACCACCAGCTCGCCAACCA	165-bp DNA fragment of the *rpfC*-coding sequence, used for RT-qPCR.
rpfF-F<br>*rpfF*-R	ATTGAAGAACCGCAGCGTGAGG<br>AGTACCACGTGTGGTGCCAGCA	170-bp DNA fragment of the *rpfF*-coding sequence, used for RT-qPCR.
hrpG-F<br>*hrpG*-R	GCACACGGATCGGCGTTTCT<br>GCAACTGGCGTCGAAGACCA	168-bp DNA fragment of the *hrpG*-coding sequence, used for RT-qPCR.

hrpX-F<br>*hrpX*-R	CCTATACGAGCAGGATGCTGGTT<br>GCGTCTTCGGCCTCTTCTTGATAT	169-bp DNA fragment of the *hrpX*-coding sequence, used for RT-qPCR.
bfd-F<br>*bfd*-R	GGTGCAATTGGTTCCGGTCC<br>AATTGGACCCACAGCCGGTG	162-bp DNA fragment of the *bfd*-coding sequence, used for RT-qPCR.
0778-F<br>0778-R	TCCTTCTTCCAGCCCTTCGC<br>CATTGGTGGTGCGGATCAGA	173-bp DNA fragment of the *XOCgx_0778*-coding sequence, used for RT-qPCR.
0420-F<br>0420-R	ATGAAGCGTGCAATTGTGCTGC<br>CGCTGCGTCTTCCACCTTCT	126-bp DNA fragment of the *XOCgx_0420*-coding sequence, used for RT-qPCR.
1445-F<br>1445-R	AACAAGGAACGGCCGGTTGT<br>CGCCCGGGAAATGGATATCG	184-bp DNA fragment of the *XOCgx_1445*-coding sequence, used for RT-qPCR.
1554-F<br>1554-R	GTCGGTTTCGAAGGCGAGCT<br>TCAGCGTGGACGTGGGTGAT	164-bp DNA fragment of the *fadE*-coding sequence, used for RT-qPCR.
2726-F<br>2726-R	AGAACCACCCTGGAGAACCTGA<br>ATGGATGGTGGTCGACGACG	195-bp DNA fragment of the *XOCgx_2726*-coding sequence, used for RT-qPCR.
3051-F<br>3051-R	CGTCCGCTGGCACAACTTGT<br>AGCGAGCCCAGACCAAGCAA	168-bp DNA fragment of the *gumE*-coding sequence, used for RT-qPCR.
3121-F<br>3121-R	GCTTCGGTGTCGCAATCGCT<br>TGCATTGGTGGTGGTGTCATCT	185-bp DNA fragment of the *pstA*-coding sequence, used for RT-qPCR.
4080-F<br>4080-R	TAACGCGCTCTATCTGCCAGG<br>TGCTGGGTCATGGTCACCTG	175-bp DNA fragment of the *XOCgx_4080*-coding sequence, used for RT-qPCR.
4385-F<br>4385-R	CAACACCGCGCATTCCCAAG<br>GAAGTGGCCGAACTACCCGA	165-bp DNA fragment of the *XOCgx_4385*-coding sequence, used for RT-qPCR.
2651-F<br>2651-R	ATTACACCGGCGTCACTGTC<br>ATAGAAGCCACGGCACAGGT	152-bp DNA fragment of the *feoB*-coding sequence, used for RT-qPCR.
0022-F<br>0022-R	GCCGCGCGCTGTCTTTGTTA<br>TCGGCCGCCGGATAGTGATA	160-bp DNA fragment of the *XOCgx_0022*-coding sequence, used for RT-qPCR.
2486-F<br>2486-R	CAATCTACCGACCGCTGCCA<br>AAGCTCGCGCTGCAGGTTCT	160-bp DNA fragment of the *flgM*-coding sequence, used for RT-qPCR.
3136-F<br>3136-R	GTCGGCCCGCTTGAAGGTAT<br>CGTCGAAGCGGTAATAGGCC	203-bp DNA fragment of the *XOCgx_3136*-coding sequence, used for RT-qPCR.
1907-F<br>1907-R	ACACCGGTGCCGATTTACCG<br>CGCGATTGACGTCGATGACC	168-bp DNA fragment of the *hutG*-coding sequence, used for RT-qPCR.
1557-F<br>1557-R	TGAGCGTGAAGATTTCATGCG<br>GACAGATCGTACGGAACCGGA	198-bp DNA fragment of the *XOCgx_1557*-coding sequence, used for RT-qPCR.
hrpB-F<br>*hrpB*-R	CCGTTGTTGCCGCAGATTCG<br>GCGGCTCCAGCATCACGATC	151-bp DNA fragment of the *hrpB*-coding sequence, used for RT-qPCR.
xopN-F<br>*xopN*-R	TGCGGGCATCCATGAGATCG<br>GATTGCAAGTGGCTGGCCGA	171-bp DNA fragment of the *xopN*-coding sequence, used for RT-qPCR.
hrcQ-F<br>*hrcQ*-R	ATGCGGCTTCGTGACACATT<br>CATTCCCGCGATCGAAACGC	166-bp DNA fragment of the *hrcQ*-coding sequence, used for RT-qPCR.
hrcT-F<br>*hrcT*-R	TGTCGCTGTTGACCCTGCTG<br>CCGTCTGGATCTTCACCAGC	177-bp DNA fragment of the *hrcT*-coding sequence, used for RT-qPCR.
hrpD6-F<br>*hrpD6*-R	TCGATGCAATGACCGATGCG<br>CATGCAGGACGCTGAAGTCG	186-bp DNA fragment of the *hrpD6*-coding sequence, used for RT-qPCR.

xopW-F<br>*xopW*-R	ATTCCGGCAGCTCCGACG<br>GGCCTACAGGACGAGACTCA	180-bp DNA fragment of the *xopW*-coding sequence, used for RT-qPCR.
gumD-F<br>*gumD*-R	ATGCTTTTGGCAGACTTGAG<br>AATCGCCAGCCGATACG	189-bp DNA fragment of the *gumD*-coding sequence, used for RT-qPCR.
fliS-F<br>*fliS*-R	ATGTACGGTTCCAATCGTCAAT<br>AAGTGACCGACGATTGCGC	209-bp DNA fragment of the *fliS*-coding sequence, used for RT-qPCR.
fliD-F<br>*fliD*-R	TACCGTGGTGTCTACCCTGG<br>CATCGGTCGGCACCGTGGCC	197-bp DNA fragment of the *fliD*-coding sequence, used for RT-qPCR.
fliC-F<br>*fliC*-R	TGAACGCTCAGCGTAACCTC<br>ACAGCGAGATGCCGTCGTTG	189-bp DNA fragment of the *fliC*-coding sequence, used for RT-qPCR.
pKG-F<br>pKG-R	CCAAGCTT ATGCAGGATGTTTCAGGAGGTC<br>GCTCTAGA TCACACCCCGGGTCGCGCCGATG	1137-bp DNA fragment containing the full length *rpfG* gene. Used for constitutive expression tests.
pKX-F<br>pKX-R	CCAAGCTT ATGATCCTTTCGACCTACTTTGCAGC<br>GCTCTAGA TTACCGTTGCAAGGTTTCCATCGG	1431-bp DNA fragment containing the full length *hrpX* gene. Used for constitutive expression tests.
LXOCgx_0458-F<br>LXOCgx_0458-R	CGGGATCC TCGATCGCCTTGACAGTAATC<br>GCTCTAGA GAGGCTATAGCCGGGCGTGG	437-bp DNA sequence upstream of XOCgx_0458, used for construction of XOCgx_0458 deletion mutant.
RXOCgx_0458-F<br>RXOCgx_0458-R	GCTCTAGA TTGCTCGAGTTGATGGTGGCGTG<br>CCAAGCTT TGCATGGGTGTGATTCGGTGA	354-bp DNA sequence downstream of XOCgx_0458, used for construction of XOCgx_0458 deletion mutant.
LXOCgx_1016-F<br>LXOCgx_1016-R	CGGAATTC ACGGTGTGCACCAGGTTGTG<br>GCTCTAGA GCCGCCAATACTAGACCATC	443-bp DNA sequence upstream of XOCgx_1016, used for construction of XOCgx_1016 deletion mutant.
RXOCgx_1016-F<br>RXOCgx_1016-R	GCTCTAGA GCACCGCGCTGAGGGCCCCC<br>CCAAGCTT CATCTTGTGACCGGCCGACA	342-bp DNA sequence downstream of XOCgx_1016, used for construction of XOCgx_1016 deletion mutant.
LXOCgx_2380-F<br>LXOCgx_2380-R	CGGGATCC TCAGCTCCCCACGTGCACGC<br>GCTCTAGA AAACGTGGAAAGCGGGTGAA	471-bp DNA sequence upstream of XOCgx_2380, used for construction of XOCgx_2380 deletion mutant.
RXOCgx_2380-F<br>RXOCgx_2380-R	GCTCTAGA TTGGGGCCCTCTCCAGCAAC<br>CCAAGCTT AGCTGGTTCCTGGCCTTGAG	331-bp DNA sequence downstream of XOCgx_2380, used for construction of XOCgx_2380 deletion mutant.
LXOCgx_2451-F<br>LXOCgx_2451-R	CGGGATCC TTCCAGCGCCTGGATCATCA<br>GCTCTAGA GCGCGGGCCTAAAGCTCAAT	468-bp DNA sequence upstream of XOCgx_2451, used for construction of XOCgx_2451 deletion mutant.
RXOCgx_2451-F<br>RXOCgx_2451-R	GCTCTAGA ACGTTCCACAAGAGGCTTATC<br>CGAAGCTT ACGGGGCGATCTTCTTGTAC	374-bp DNA sequence downstream of XOCgx_2451, used for construction of XOCgx_2451 deletion mutant.
LXOCgx_2936-F<br><br>LXOCgx_2936-R	CGGGATCC<br>AATTCGTCTTCCTCCATGCGCCGGTAGA<br>GCTCTAGA GGCGGTAGACTATACCGTAC	502-bp DNA sequence upstream of XOCgx_2936, used for construction of XOCgx_2936 deletion mutant.
RXOCgx_2936-F<br>RXOCgx_2936-R	GCTCTAGA ATGATTGCCCTGCTCCGCTCATT<br>CCAAGCTT AGCTGGCCCTGCGCCTGCAG	336-bp DNA sequence downstream of XOCgx_2936, used for construction of XOCgx_2936 deletion mutant.
LXOCgx_3699-F<br>LXOCgx_3699-R	CGGGATCC ATAGCGGAATATCCGCATCGC<br>GCTCTAGA GGGCGGCGGTCTGCAGGGGG	422-bp DNA sequence upstream of XOCgx_3699, used for construction of XOCgx_3699 deletion mutant.
RXOCgx_3699-F<br>RXOCgx_3699-R	GCTCTAGA TCCCGAAACAGCACTGCCCCG<br>CCAAGCTT TGTCTGGATGCGGCGGTCGA	331-bp DNA sequence downstream of XOCgx_3699, used for construction of XOCgx_3699 deletion mutant.
LXOCgx_3726-F<br>LXOCgx_3726-R	CGGGATCC GTGCATGGATCACGATGCTGGCAA<br>GCTCTAGA GTGATCGGCTCGGTCAACGG	468-bp DNA sequence upstream of XOCgx_3726, used for construction of XOCgx_3726 deletion mutant.
RXOCgx_3726-F<br>RXOCgx_3726-R	GCTCTAGA GCAAGGCGCGCATGGTTGCA<br>CCAAGCTT TTCCACAGCCAAGCCCGCCA	363-bp DNA sequence downstream of XOCgx_3726, used for construction of XOCgx_3726 deletion mutant.

LXOCgx_4067-F<br>LXOCgx_4067-R	CGGGATCC ACTGGCCGTTCTTCCGCACC<br>GCTCTAGA GGTCGCCGTTGCTTTGCCGT	446-bp DNA sequence upstream of XOCgx_4067, used for construction of XOCgx_4067 deletion mutant.
RXOCgx_4067-F<br>RXOCgx_4067-R	GCTCTAGA CACCGGCCATATGCGCCCGC<br>CCAAGCTT CGGACAGCGCCTGGTTGTGG	365-bp DNA sequence downstream of XOCgx_4067, used for construction of XOCgx_4067 deletion mutant.

§The underlined sequences indicate the restriction sites for *Bam*HI, *Eco*RI, *Hin*dIII, *Xba*I, and *Xho*I, respectively.
